# Physiological and transcriptome analysis reveal molecular mechanism in *Salvia miltiorrhiza* leaves of near-isogenic male fertile lines and male sterile lines

**DOI:** 10.1186/s12864-019-6173-4

**Published:** 2019-10-26

**Authors:** Ruihong Wang, Han Jiang, Ziyun Zhou, Hongbo Guo, Juane Dong

**Affiliations:** 1 College of Life Sciences, Northwest A&F University, Yangling, 712100 China; 2 College of Chemistry and Pharmacy, Northwest A&F University, Yangling, 712100 China

**Keywords:** *Salvia miltiorrhiza*, Male sterile mutants, Active ingredients, Transcriptome, Chloroplast structure, Photosynthetic characteristics, Phenylpropanoid pathway

## Abstract

**Background:**

Our previous study finds that male sterility in *Salvia miltiorrhiza* could result in stunted growth and reduced biomass, but their molecular mechanisms have not yet been revealed. In this article, we investigate the underlying mechanism of male sterility and its impact on plant growth and metabolic yield by using physiological analysis and mRNA sequencing (RNA-Seq).

**Results:**

In this study, transcriptomic and physiological analysis were performed to identify the mechanism of male sterility in mutants and its impact on plant growth and metabolic yield. Through Gene Ontology (GO) and Kyoto Encyclopedia of Genes and Genomes (KEGG) analysis, it is found that the pathways are mainly enriched in processes including organ development, primary metabolic process and secondary metabolic process. Physiological analysis show that the chloroplast structure of male sterile mutants of *S. miltiorrhiza* is abnormally developed, which could result in decrease in leaf gas exchange (*A*, *E* and *g*_s_), chlorophyll fluorescence (F_v_, F_m_ and F_v_/F_m_), and the chlorophyll content. Expression level of 7 differentially expressed genes involved in photosynthesis-related pathways is downregulated in male sterile lines of *S. miltiorrhiza*, which could explain the corresponding phenotypic changes in chlorophyll fluorescence, chlorophyll content and leaf gas exchange. Transcriptomic analysis establishes the role of disproportionating enzyme 1 (*DPE1*) as catalyzing the degradation of starch, and the role of sucrose synthase 3 (*SUS3*) and cytosolic invertase 2 (*CINV2*) as catalyzing the degradation of sucrose in the *S. miltiorrhiza* mutants. The results also confirm that phenylalanine ammonialyase (*PAL)* is involved in the biosynthesis of rosmarinic acid and salvianolic acid B, and flavone synthase (*FLS)* is an important enzyme catalyzing steps of flavonoid biosynthesis.

**Conclusions:**

Our results from the physiological and transcriptome analysis reveal underlying mechanism of plant growth and metabolic yield in male sterile mutants, and provide insight into the crop yield of *S. miltiorrhiza*.

## Background

Male sterility could be the result of abnormal development of stamens of flowering plants, which cannot produce normal pollen, but their pistils can still develop normally and receive pollen and fertilize [1, 2]. As we know, the male sterile could be divided several types: cytoplasmic male sterility (CMS), genic male sterility (GMS) and thermo-photo sensitive genic male sterile (TPSGMS), which are found in crops such as *Zea mays*, *Oryza sativa*, *Brassica napus* and *Triticum aestivum*, etc. [1, 3–6]. Shu et al. discovered a natural male sterile mutant of *Salvia miltiorrhiza* in 2002 [7]. Through continuous backcross and testcross in 2006–2009, we established that this male sterile mutant of *S. miltiorrhiza* was GMS type [7, 8]. Male sterility systems have been widely used in crops such as maize, wheat, and rice [4, 9, 10]. Male sterility plays an extremely important role in heterosis utilization and hybrid seed yield of crops and its use has successfully increased crop yield [11–13]. *S. miltiorrhiza* is generally used as a medicinal plant and has been widely used to treat cardiovascular and cerebrovascular diseases, hyperlipidemia, and acute ischemic stroke [14–18]. There are many traditional Chinese medicines such as Fufang Danshen Tablets and Danshen injections, etc., which contain active ingredients extracted from *S. miltiorrhiza* [19]. Due to the increasing demand in recent years, its planting area has expanded rapidly. The *S. miltiorrhiza* cultivation is, however, affected by the factors such as climate and region, which lead to unstable quality of active ingredients. Since the discovery of the male sterile mutants of *S. miltiorrhiza*, we have created and developed near-isogenic male fertile lines (MF) and near-isogenic male sterile lines (MS) through multiple consecutive testcrosses and backcrosses [20]. It promises possibility to achieve stable and controllable quality of active ingredients.

Since the discovery of the male sterile mutants of *S. miltiorrhiza* in 2002, our team has done some research work [7, 20]. Researchers have studied the development and biological properties of pollen. The markers tightly linked to drought stress genes have been identified and the genetic linkage map between male fertile lines and male sterile lines of *S. miltiorrhiza* has been constructed by amplified fragment length polymorphism (AFLP). The results also find that male sterility in *S. miltiorrhiza* could result in stunted growth and reduced biomass. At present, the research on male sterility mainly focuses on the regulation and restoration of fertility, but little attention has been paid to the effect on plant growth and metabolic yield. The molecular mechanism of plant growth and metabolic yield in the male sterile mutants of *S. miltiorrhiza* has not yet been studied.

The revelation of underlying mechanisms of plant growth and metabolic yield is very important to help the crop improvement. Here, we analyse the primary metabolism in leaves and secondary metabolism (active ingredients) in leaves, flowers and roots of *S. miltiorrhiza* between male fertile lines and male sterile lines. It is shown that primary metabolism in male sterile mutants is decreased but secondary metabolism is boosted. In order to elucidate the molecular mechanism, we performed the transcriptome and physiological analysis of the *S. miltiorrhiza* leaves of male fertile lines and male sterile lines. Our results from the physiological and transcriptomic analysis reveal underlying mechanism of plant growth and metabolic yield in the plant leaves, and provide insight into crop yield of *S. miltiorrhiza*.

## Methods

### Plant materials

This work used *S. miltiorrhiza* leaves of near-isogenic male fertile lines and near-isogenic male sterile lines as experimental materials, which were collected from a botanical garden of *S. miltiorrhiza* at Northwest A&F University in Yangling, Shaanxi Province, China. The two plant materials (MF *S. miltiorrhiza* and MS *S. miltiorrhiza*) used in this paper had been grown under same growth conditions and for same growth years. The other phenotypes of tissues were presented in Additional file 1: Figure S1A-B. The leaves of MF and MS were processed under sterile conditions, immediately frozen and stored in liquid nitrogen at − 80 °C before the RNA isolation. Leaf subcellular structures and features were observed by transmission electron microscopy (TEM).

### Determination of primary metabolites

Polysaccharide, starch, sucrose and proteins were identified and estimated by an anthrone-sulfuric acid assay, a perchloric acid hydrolysis, a resorcinol-based assay and a Coomassie Brilliant Blue G*-*250-based assay.

### Determination of secondary metabolites

In this study, we collected 5 whole plants, deactivated their enzymes at 105 °C for 15 min, and then dried them to constant weight at 45 °C. The dried leaves of *S. miltiorrhisa* were weighed about 0.1 g, and 4 ml 70% methanol aqueous solution was added. The material stood overnight in the methanol aqueous solution, and ultrasonic extraction was applied for 45 min and the extract was separated by centrifugation. The supernatant was taken for determination of total phenolic, total flavonoid and phenolic acids.

Total phenolic was determined using a modified method [21]. Test samples (1.0 mL) were mixed with 1 mL Folin-Ciocalteu and incubated at room temperature for 3 min. After the addition of 4.0 mL of 7.5% Na_2_CO_3_ and 4.0 mL distilled water, the reaction tubes were incubated for 1 h at 40 °C and the absorbance readings were taken at 765 nm. The measurements were done with a calibration curve plotted against gallic acid to establish the content of total phenolic in extracts, whose results are shown as milligrams of gallic acid equivalents per gram of dry extract. The curve eq. Y = 0.0128 X + 0.0001 (R^2^ = 0.9994) is used, where Y is absorbance and X total phenolic content.

Total flavonoid content was measured using a modified aluminums chloride colorimetric assay [22]. Test samples (1.0 mL) were mixed with 6.4 mL 30% ethanol, 0.3 mL 0.5 M NaNO_2_ and 0.3 mL 0.3 M AlCl_3_ and kept in the dark for 5 min at room temperature. After the addition of 2.0 mL of 1 M NaOH, the absorbance readings were taken at 506 nm. The same measurements and calculations were done to establish total flavonoid content. And the gallic acid curve equation is shown as Y = 0.0007 X + 0.0071 (R^2^ = 0.9991).

The extraction of phenolic acids was done according to the protocol for total phenolic and total flavonoid. The quantitative and qualitative analysis of the phenolic acids were carried out according to reference procedures [23]. All chromatographic isolation was performed by using HPLC system equipped with an autoinjector, a UV detector, and LC-solution software (Waters, UK). The column type, column temperature, mobile phase flow rate, mobile phase solvent ratio and program settings are set according to the reference. The injection volume was 10 μl. Spectral data were collected at 280 nm in the whole run. Sample and mobile phase filtration were done at a 0.22 μm filter before a HPLC injection. Analysis of each extract was conducted in triplicate.

### RNA extraction, library preparation and mRNA-seq

Total RNA was extracted with an RNA pure Plant kit (Tiangen Biotech Co., Ltd., Beijing, China). The total RNA concentration, RIN value, 28S/18S and fragment size were determined using an Agilent 2100 Bioanalyser (Agilent Technologies Co. Ltd., Santa Clara, CA, USA) and an Agilent RNA 6000 Nano Kit. The purity measurement of the samples was made using an ultraviolet spectrophotometer NanoDrop™. After the isolation and fragmentation of the total RNA, the eukaryotic mRNA was extracted by using Oligo (dT) coupled to magnetic beads. The preparation of cDNA libraries was based on the PCR method described in the following procedure. The first strand cDNA was manufactured from the fully spliced mRNA as a template, and then a second strand reaction system was used to make double-stranded cDNA. The second strand cDNA was synthesized and purified using a system kit. When the cDNA ends were repaired, an ‘A’ nucleobase hybridized to the 3᾽ ends of the cDNA. Then, the cDNA fragments of different sizes were selected and then amplified. The qualities of the amplified cDNA libraries were tested. Finally, the sequencing was run on an Illumina HiSeq™ 2500 platform.

### FPKM calculation and DESeq2 differential expression analysis

We used the alignment package Bowtie2 (v2.2.5) to align clean reads back to reference genomes [24]. Then the gene expression level of every sample was calculated by RSEM (v1.2.12), a software package designed to estimate gene and isoform expression levels from RNA*-*Seq data [25]. Fragments per kilobase per million (FPKM) mapped reads values were calculated by mapping the reads to fragments, which could be used to quantify the abundance of the transcripts and so to analyse differential gene expression in the samples. Differential expression analysis was performed with DESeq2 package [26]. DEGs were selected according to *p*-value (*p* ≤ 0.05) and fold change analysis (FC ≥ 2.00).

### GO and KEGG enrichment analysis of DEGs

According to the official classification categories and based on the GO and KEGG annotation results [27], the DEGs are classified into functional and biological pathways, and the R function phyper is used for enrichment analysis. A *p*-value of 0.05 (*p* ≤ 0.05) implies that the accepted false discovery rate (FDR) is 5%.

### RT-qPCR validation and DEGs analysis

To validate internal control genes for expression analysis, the ABI StepOnePlus™ RT-PCR System (Applied Biosystems, USA) was used to perform RT-qPCR and investigate differential expression of gene sets. GenScript Real-time PCR online software was adopted to build sequencing primers, shown in Additional file 2: Table S1. RT-qPCR were carried out using the PrimeScript™ RT reagent Kit and SYBR Green™ Premix Ex Taq™ to transcribe RNA into complementary DNA (cDNA) (Takara Biotech Co., Ltd., Japan) with β-actin used as an internal control. Three independent technical replicates and three biological replicates for each sample were used to validate the performance of RT-qPCR.

### Determination of leaf photosynthesis and chlorophyll content

The photosynthetic parameters were determined between 08:00 am to 12:00 am by the portable photosynthesis system (LI-6400, LI-COR Inc., USA) [28]. Fluorescence kinetic values of chlorophyll were determined by FluorCam fluorometer acquisition system (PDA-100 Heinz Walz, Germany). Chlorophyll fluorescence analysis was used to assess the photosynthetic performance of *S. miltiorrhiza*. Before the measurements, the leaves were kept in the dark for 30 min using cuvettes on ice. A 5 s light pulse at 400 μmol m^− 2^ s^− 1^ was employed.

In this study, about 0.1 g (fully expanded second leaf blade) of the fresh sample was extracted with 80% acetone, and then the chlorophyll content of the leaves was determined by an ultraviolet spectrophotometer. Absorbance of the chlorophyll extractions and blank (80% acetone) were measured at 663 nm and 645 nm. Finally, chlorophyll a, b and total chlorophyll were calculated by Arnon᾽ equations.

### Statistical analysis

Each experiment was carried out in triplicates. The results were shown as means ± standard error and dealt with using SPSS (version 21.0). ANOVA and Tukey᾽s Honestly Significant Difference were used to determine significant differences among the samples. A 0.05 significance level was considered.

## Results

### Male sterility inhibiting primary metabolism

**Fig. 1:**
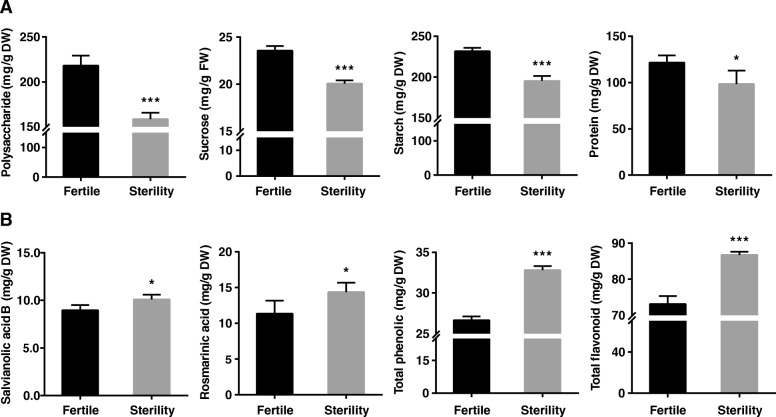
Determination the content of primary metabolites and secondary metabolites between MF and MS in *S. miltiorrhiza* leaves. **a** Polysaccharide, sucrose, starch and protein. **b** Salvianolic acid B, rosmarinic acid, total phenolic and total flavonoid. Vertical bars indicate standard errors (SE) of the mean (*n* = 3). Asterisk represents significant differences between MF and MS (^∗^*P* < 0.05, ^∗∗^*P* < 0.01, ^∗∗∗^*P* < 0.001, *t*-test)

Since the discovery of the natural male sterile mutants of *S. miltiorrhiza* in 2002, a lot of laboratory work has been done to create and develop near-isogenic lines. After many generations of testcrossing, backcrossing and genotyping, our team obtained genetically stable near-isogenic male fertile (MF) and near-isogenic male sterile (MS) lines. Previous results showed that the height of MF *S. miltiorrhiza* was 1.71 times higher than that of MS ones. In this study, we measured the content of primary metabolites including polysaccharide, sucrose, starch, and protein (Fig. 1a). The results showed very significant differences in the content of the primary metabolites in MF and MS *S. miltiorrhiza* leaves, and the MF *S. miltiorrhiza* leaves had higher content of primary metabolites than those in MS *S. miltiorrhiza* leaves. Specifically, the content of polysaccharide, sucrose, starch and proteins were respectively 27.30, 14.91, 15.72 and 18.90% higher in MF plants than in MS plants.

### Male sterility promoting secondary metabolites

In previous studies, some phenolic acids have been perceived as the major active ingredients of the aqueous extracts of *S. miltiorrhiza*, including rosmarinic acid and salvianolic acids B. In all analysed extracts, salvianolic acid B and rosmarinic acid were most plentiful, both recognized as antioxidants. These compounds are cause of the antioxidant activity in both root and leaf extracts, indicating that they are present in the whole plant of *S. miltiorrhiza*, regardless of their tissue type and location. The flavonoids and polyphenols could also play important roles in antioxidant activity, since they are particularly rich in *S. miltiorrhiza* leaves [29]. As a result, the *S. miltiorrhiza* leaf extract can be used as a powerful herbal antioxidant activity [30]. As we know, the leaf biomass of *S. miltiorrhiza* makes up a significant fraction of the whole plant. Nevertheless, during the harvest, the leaves of *S. miltiorrhiza* are disposed as wastes. The results indicated that the leaves of *S. miltiorrhiza* could make a natural material for drug, nutritious foods, cosmetics and other industries.

In this paper, we identified some of the above-mentioned active ingredients in MF and MS *S. miltiorrhiza* leaves (Fig. 1b). The results show that the content of these secondary metabolites was higher in MS leaves than in MF leaves. In Fig. 1b, we have found that the relative content of salvianolic acid B, rosmarinic acid, total phenolic and total flavonoid are respectively 12.62, 23.59, 18.55, and 23.06% higher in MS leaves than in MF leaves. We have also measured the active ingredients of salvianolic acid B and rosmarinic acid in other tissues (Additional file 1: Figure S1 C, D). The results suggest that the content of salvianolic acid B and rosmarinic acid are higher in MS flowers and roots than in MF flowers and roots. In flowers, the content of salvianolic acid B and rosmarinic acid in male sterile lines is 17.96 and 21.18% higher than in male fertile lines, respectively. And in the roots, the content of salvianolic acid B and rosmarinic acid in male sterile lines is 18.71 and 32.81% higher than in male fertile lines, respectively. In this article, we *have* determined the compositions of the tanshinones (including tanshinone I, tanshinone II-A, dihydrotanshinone and cryptotanshinone) in the flowers, leaves and roots of *S. miltiorrhiza*. It has been found that tanshinones are detected only in the roots (Additional file 1: Figure S1-E), while no tanshinones are detected in the flowers and leaves, indicating that tanshinones are tissue specific. In the roots, the content of tanshinone I, tanshinone II-A, dihydrotanshinone and cryptotanshinone in male sterile lines are 82.58, 82.06, 72.58 and 110.97% higher than in male fertile lines, respectively. The results indicate that the active ingredients in the MS tissues are more abundant than in the MF tissues. In all, we infer that male sterile mutants have the primary metabolism inhibited but have the secondary metabolism promoted in leaves. However, the underlying mechanisms of plant growth and metabolic yield in the male sterile mutants are still unclear.

### The analysis of RNA-seq data

To explore the molecular mechanisms of plant growth and metabolic yield in male sterile *S. miltiorrhiza* leaves, we used transcriptomics for further research. Six libraries (male fertility: F1, F2 and F3; male sterility: S1, S2 and S3) were prepared, and the preparation of the library for each sample was repeated 3 times. We used the Illumina HiSeq™ 2500 platform to sequence the libraries. The transcriptomic profile of filtration, reads, ribosomal alignment, and genes coverage are shown in Additional file 3-7: Table S2, Table S3, Table S4, Figures S2 and Figure S3, respectively. In Additional file 3: Table S2, we find that the clean reads for each sample are more than 3.0 Gb. The results in Additional file 4: Table S3 show that the Q20 > 90%, Q30 > 90% and *N* < 5% after filtering. In Additional file 5: Table S4, the statistics show less than 5% ribosomal RNA sequences of each sample are aligned. The results in Additional file 6: Figure S2 and Additional file 7 Figure S3 find that the majority of genes coverage is in the range of 80 and 100% and most raw reads was classified as clean reads. These results demonstrate that the transcriptional profiling data are reliable for further analysis. After data filtering, reads were aligned to the reference genome and the statistical results are shown in Table 1. The ratio of mapped reads to the reference genome were 95.04, 91.35, 92.71, 91.3, 94.66, and 90.84%, respectively.

**Table 1:** The statistics of reference genome alignment results

Sample	All Reads Num	Mapped Reads	Unmapped Reads
F1	10,219,982	9,713,429 (95.04%)	506,553 (4.96%)
F2	10,465,470	9,560,268 (91.35%)	905,202 (8.65%)
F3	44,454,092	41,211,575 (92.71%)	3,242,517 (7.29%)
S1	10,038,332	9,173,391 (91.38%)	864,941 (8.62%)
S2	10,506,634	9,946,024 (94.66%)	560,610 (5.34%)
S3	46,055,644	41,835,694 (90.84%)	4,219,950 (9.16%)

Note: The three biological replicates of male fertility are F1, F2 and F3, and the three biological replicates of male sterility are S1, S2 and S3

### Analysis of GO, KEGG classification and enrichment of DEGs

**Fig. 2:**
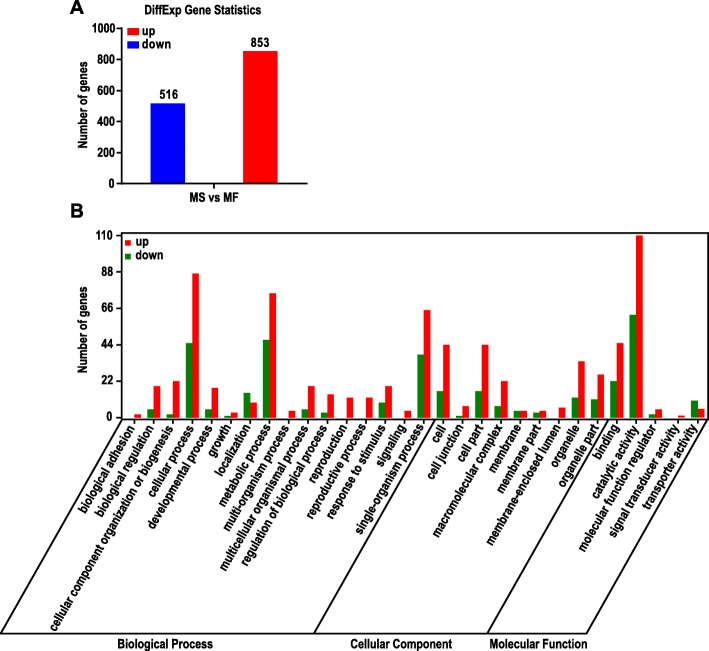
The number (**a**) and GO functional annotation and classification (**b**) of differentially expressed genes between MF and MS of *S. miltiorrhiza*. In the figure, MF is used as a control group. Green represents downregulated and red represents up-regulated. **a** The number of differentially expressed genes. **b** The abscissa was the GO classification, which was divided into three categories: biological process, cellular component and molecular function. The left side of the ordinate was the number of genes. The figure showed the enrichment of upregulated genes and downregulated genes in the secondary functions of GO

**Fig. 3:**
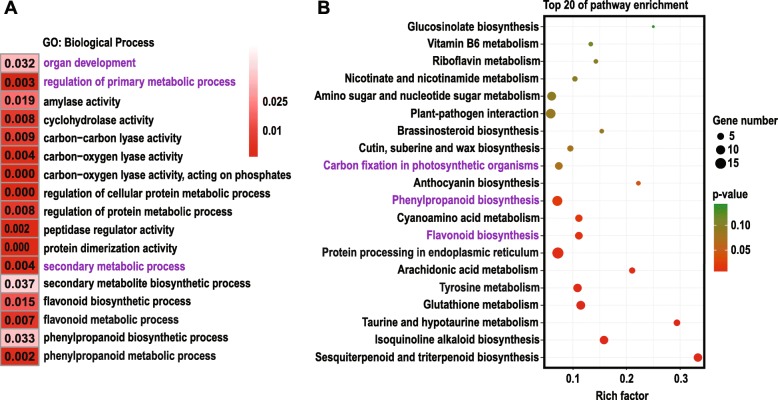
GO (**a**) and KEGG (**b**) enrichment between MF and MS of *S. miltiorrhiza*. In the figure, MF is used as a control group. **a** GO (biological process) enrichment of differentially expressed genes. **b** The KEGG-enriched bubble map of differentially expressed genes. The biological processes and metabolic pathways we are interested in are shown in purple

After parameter screening and optimization (*p* < 0.05, |log_2_FC| > 1), GO analysis identified a total of 1369 DEGs between MF and MS lines. Figure 2a shows a total of 853 differentially expressed genes are upregulated and 516 downregulated. Through GO analysis of DEGs, 30 significantly represented terms are displayed in Fig. 2b. The results show that the metabolic process, cellular process and single-organism process terms were the most represented GO terms in the biological process category. Cell, cell part, organelle, organelle part and macromolecular complex are the most shared terms in the cellular component category. Catalytic activity and binding are significantly represented terms in the molecular function category. Through the deep mining analysis of the data, we selected several GO enrichment pathways that were represented in the data, as shown in Fig. 3a. It has been found that these pathways were mainly enriched in processes related to organ development, primary metabolic process and secondary metabolic process.

According to the KEGG orthology classification, DEGs have been annotated into 90 pathways (Additional file 8: Table S5) and divided into five categories (Additional file 9: Figure S4). Assignment of DEGs to metabolic pathways provides insight into the biological functions and gene interactions and we find that the most represented category was metabolism. The results show that the metabolism category is the most annotated, including carbohydrate metabolism, biosynthesis of other secondary metabolites, etc. The bubble map of the DEG pathway enrichment analysis (only the top 20 metabolic pathways shown in Fig. 3b) demonstrates that the metabolic pathways with significant enrichment are phenylpropanoid biosynthesis pathway, protein processing in endoplasmic reticulum pathway, flavonoid biosynthesis pathway, tyrosine metabolism pathway and carbon fixation in photosynthetic organisms pathway. In Fig. 3, the GO and KEGG analyses suggest that their pathway enrichment patterns are basically similar, and those pathways include flavonoid biosynthesis and phenylpropanoid biosynthesis.

### Abnormal development of chloroplast structure in male sterile mutants

**Fig. 4:**
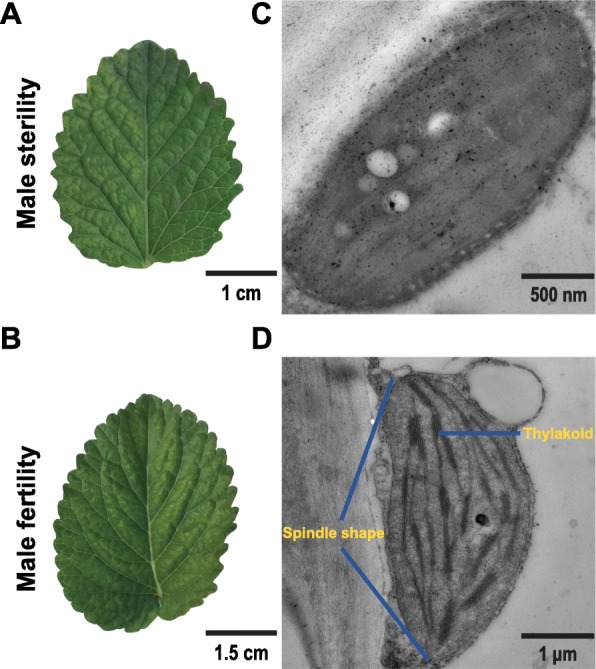
Morphological map of near-isogenic male fertile lines (MF) and near-isogenic male sterile lines (MS) of *S. miltiorrhiza* leaves. **a** Male sterile leaf, **b** male fertile leaf, **c** chloroplast subcellular structure of male sterile leaf and **d** chloroplast subcellular structure of male fertile leaf. The blue lines indicate the thylakoid and spindle shape, respectively. The scale bar of each figure was indicated in the lower right corner

Phenotype observation shows that the leaves of the male sterile lines are significantly smaller than those of the male fertile lines (Fig. 4a, b). Previous studies in the laboratory have found that the leaves of the male fertile lines are 1.46 times as long and 1.38 times as wide as those of the male sterile lines (25 samples from each line). Differences in organ development have been found in the GO annotation results of the transcriptomic data. Furthermore, the subcellular structure of leaves was observed by transmission electron microscopy. Interestingly, we find that the chloroplast structure of the two samples is significantly different, which is shown in Fig. 4c, d. The results show that the chloroplast structure of male fertile leaves is fully developed and the thylakoid structure can be clearly seen. However, thylakoid structures could not be observed in the leaves of male sterile mutants. In addition, there is no spindle-like chloroplast in the male sterile leaves. Therefore, the results suggest that the chloroplast development of male sterile leaves might be incomplete.

### Reduction of leaf gas exchange characteristics, chlorophyll fluorescence parameters and photosynthesis pigments in male sterile mutants

**Fig. 5:**
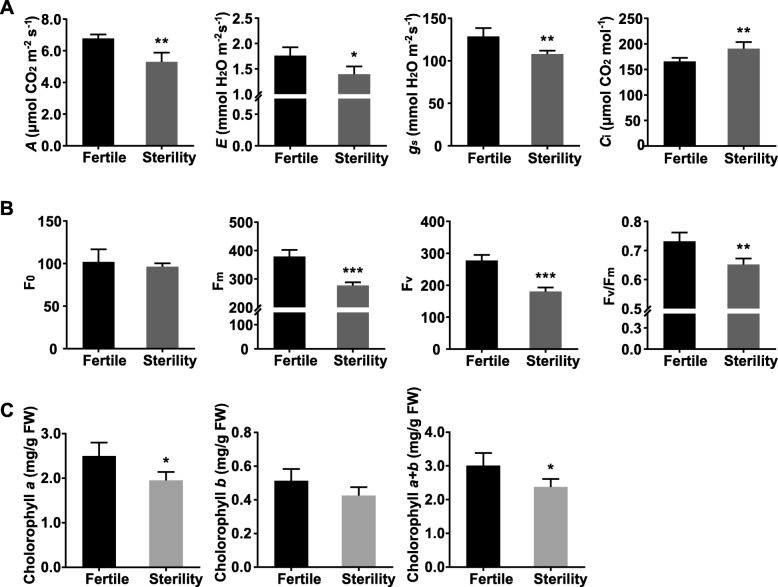
Determination of leaf gas exchange characteristics, chlorophyll fluorescence parameters and photosynthesis pigments between MF and MS in *S. miltiorrhiza* leaves. **a** Net photosynthetic rate (*A*), transpiration rate (*E*), stomatal conductance (*g*_s_) and intercellular CO_2_ concentration (*C*_i_). **b** Minimal fluorescence (F_0_), maximal fluorescence (F_m_), variable fluorescence (F_v_) and maximal quantum yield of PSII (F_v_/F_m_). **c** Chlorophyll *a* content, chlorophyll *b* content and chlorophyll *a + b* content. Vertical bars indicate standard errors (SE) of the mean (*n* = 3). Asterisk represents significant differences between MF and MS (^∗^*P* < 0.05, ^∗∗^*P* < 0.01, ^∗∗∗^*P* < 0.001, *t*-test)

In this study, we have measured the photosynthetic indicators of *S. miltiorrhiza* leaves of the male fertile lines and male sterile lines, and found that all the values of the indicators including the net photosynthetic rate (*A*), transpiration rate (*E*) and stomatal conductance (*g*_s_) of the male fertile lines are higher than those of the male sterile lines except intercellular CO_2_ concentration (*C*_i_) in *S. miltiorrhiza* (Fig. 5a). The results show that the abnormal chloroplast structure points to the reduced photosynthesis. The results also indicate that the net photosynthetic rate is positively correlated with the stomatal conductance.

Using chlorophyll fluorescence, a natural probe emitted from plants, can detect a lot of information about the state of plant growth [31]. By comparing the chlorophyll fluorescence detected from male fertile and male sterile plant leaves, it is found that there are significantly variations in F_m_, F_v_ and F_v_/F_m_ between the two lines except F_0_ (Fig. 5b), and they are positively correlated with their growth and photosynthesis. This indicates that a higher growth rate is closely related to higher PSII potential viability and higher light conversion efficiency. These studies have fully demonstrated that chlorophyll fluorescence could sensitively reflect the growth and development of plants, so it might become one of the indicators for predicting plant growth potential.

Furthermore, we have determined the photosynthetic pigment content of the *S. miltiorrhiza* leaves of male fertile and sterile lines. Our results find that the chlorophyll *a* content and chlorophyll *a + b* content of male fertile and sterile leaves are significantly different, but there is no significant difference found in chlorophyll b content in both leaves (Fig. 5c).

### DEGs related to photosynthesis and carbon fixation in photosynthetic organisms

**Fig. 6:**
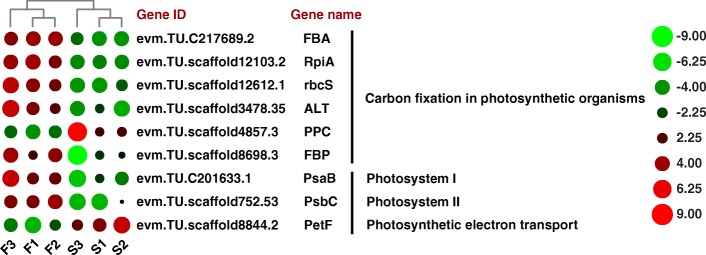
Photosynthesis and carbon fixation in photosynthetic organisms pathway between MF and MS in *S. miltiorrhiza* leaves. Green represents downregulated and red represents upregulated. FBA: Fructose-bisphosphate aldolase; RpiA: ribose 5-phosphate isomerase A; rbcS: ribulose 1,5-bisphosphate carboxylase small subunit; ALT: alanine transaminase; PPC: phosphoenolpyruvate carboxylase and FBP: fructose-1,6-bisphosphatase

To understand the incomplete development of chloroplast in leaves of MS *S. miltiorrhiza*, we have analysed the DEGs which are related to photosynthesis and carbon fixation in photosynthetic organisms (Fig. 6). We identify three photosynthesis-related genes, which include one PSI gene (*PsaB*), one PSII gene (*PsbC*) and one photosynthetic electron transport gene (*PetF*), have been differentially expressed between MF and MS lines. And we also identify other 6 genes, encoding 6 enzymes responsible for carbon fixation, which include fructose-bisphosphate aldolase (*FBA*), ribose 5-phosphate isomerase A (*RpiA*), ribulose 1,5-bisphosphate carboxylase small subunit (*rbcS*), alanine transaminase (*ALT*), phosphoenolpyruvate carboxylase (*PPC*) and fructose-1,6-bisphosphatase (*FBP*), have significantly differential expression between the MF and MS lines.

In Fig. 6, most of these genes are downregulated in male fertile lines. For example, *FBA*, *RpiA*, *rbcS*, *ALT*, *FBP*, *PsaB* and *PsbC* are downregulated in male sterile lines of *S. miltiorrhiza*. However, two genes (i.e., *PPC* and *PetF*) are upregulated in male sterile lines of *S. miltiorrhiza*. Most of these enzymes are downregulated in male sterile lines of *S. miltiorrhiza* and these results are consistent with the phenotypic changes including net photosynthetic rate, chlorophyll fluorescence, and chlorophyll content (Fig. 5).

### DEGs involved in sucrose-starch metabolism pathway between male fertile lines and male sterile lines of *S. miltiorrhiza*

**Fig. 7:**
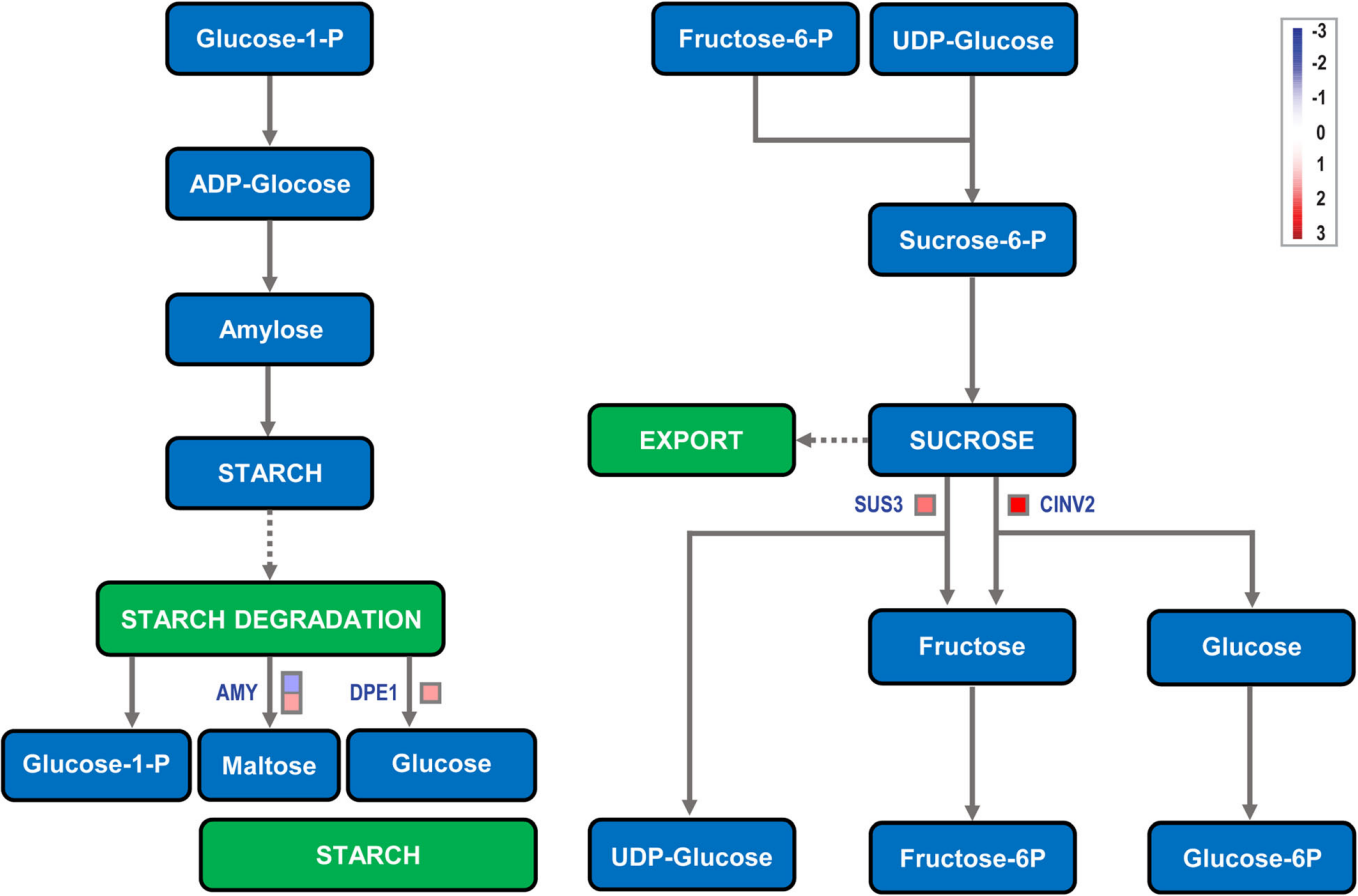
Sucrose-starch metabolism pathway between MF and MS in *S. miltiorrhiza* leaves. In the figure, MF is used as a control group. Blue represents downregulated and red represents upregulated. AMY: Alpha amylase; DPE1: Disproportionating enzyme 1; SUS3: Sucrose synthase 3; CINV2: Cytosolic invertase 2

In order to further explore the metabolic pathways, we used MapMan program to analyse the transcriptome data. MapMan is specialized in functional classification of genes in metabolic pathways and biological processes in organisms [32, 33], which is excellent at visualizing gene function classification and gene expression data. Mapping files are obtained from Mercator Automated Sequence Annotation using nucleic acid sequences of *S. miltiorrhiza* (Additional file 10: Excel S1). We find that there are significant differences (Fig. 7) in sucrose synthase 3 (*SUS3*), cytosolic invertase 2 (*CINV2*), alpha amylase (*AMY*) and disproportionating enzyme 1 (*DPE1*) in the sucrose-starch metabolism pathway. It is inferred that the relative content of sucrose and starch is lower in the male sterile mutants than in male fertile plants (Fig. 1A). In Fig. 7, the DEGs between the two lines are mainly involved in the degradation process of sucrose and starch.

### DEGs involved in phenylpropanoid metabolism pathway between male fertile lines and male sterile lines of *S. miltiorrhiza*

**Fig. 8:**
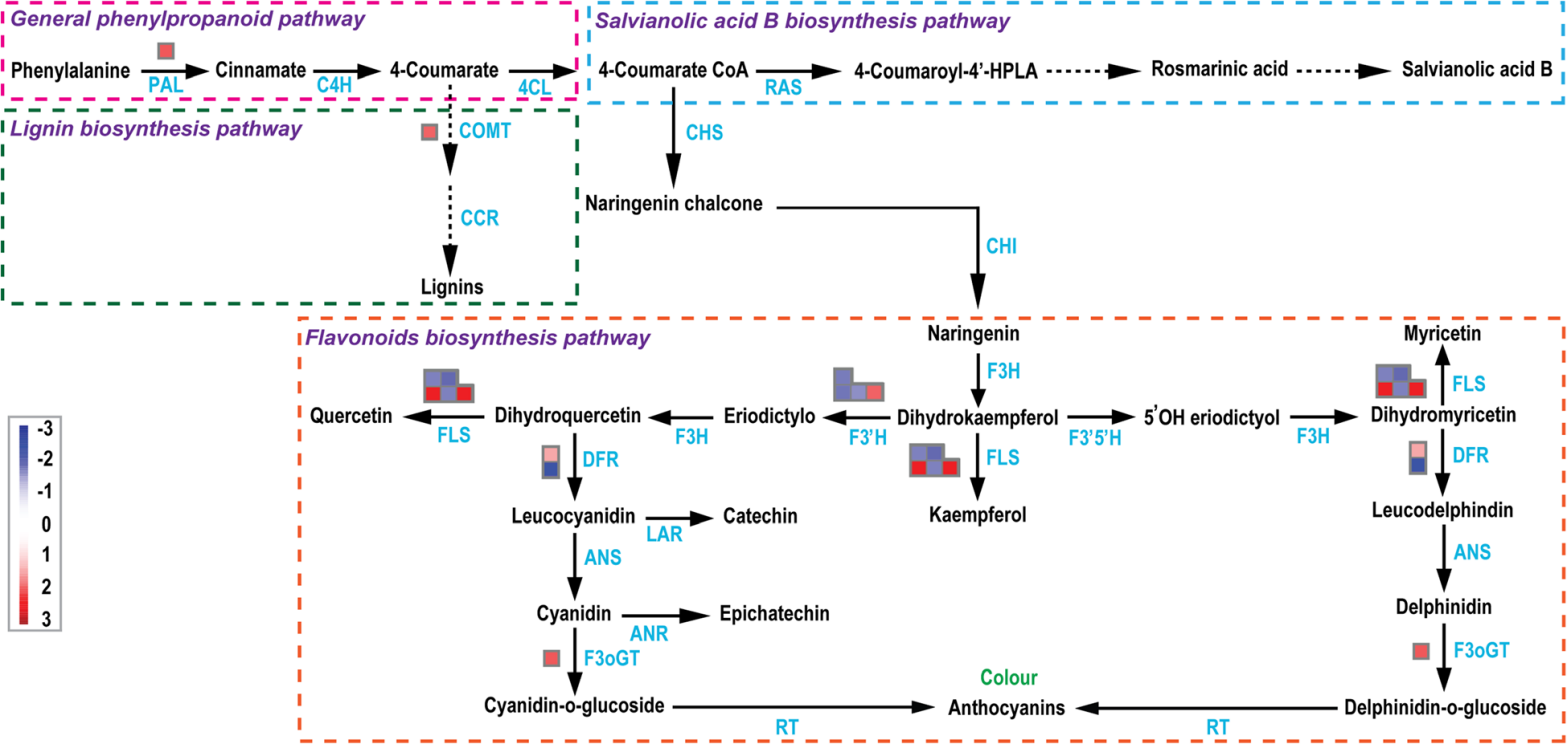
Phenylpropanoid biosynthesis pathway between MF and MS in *S. miltiorrhiza* leaves. MF is used as a control group. Blue represents downregulated and red represents upregulated. PAL: Phenylalanine ammonialyase; C4H: Cinnamate 4-hydroxylase; 4CL: 4-coumarate-CoA ligase; RAS: Rosmarinic acid synthase; CHS: Chalcone synthase; COMT: Caffeic acid O-methyltransferase; CHI: Chalcone isomerase; CCR: Cinnamoyl-CoA reductase; F3H: Flavanone 3-Hydroxylase; FLS: Flavonol synthetase; F3’5’H: Flavonoid-3′,5′-hydroxylase; DFR: Dihydroflavonol 4-reductase; F3᾽H: Flavonoid 3᾽-hydroxylase; ANS: Anthocyanidin synthase; LAR: Leucoanthocyanidin reductase; ANR: Anthocyanidin reductase; F3oGT: Flavonol 3-O-glucosyltransferase

The phenylpropanoid metabolism pathway has important physiological significance in plants. And its intermediate products and its further products are closely related to physiological activities such as differentiation of cells in plant development, resistance to pathogen infection, and formation of pigmentation [34]. We have also used MapMan program to analyse the phenylpropanoid metabolism pathway, and the results are shown in Fig. 8 and Additional file 11: Figure S5. It could be seen that there are significant differences in the key enzymes of the phenylpropanoid metabolism pathway, which include phenylalanine ammonialyase (*PAL*) in general phenylpropanoid pathway, caffeic acid O-methyltransferase (*COMT*) and ferulic acid-5-hydroxylase (*F5H*) in lignin biosynthesis pathway, and flavonoid 3᾽-hydroxylase (*F3᾽H*), flavonol synthetase (*FLS*), dihydroflavonol 4-reductase (*DFR*) and flavonol 3-O-glucosyltransferase (*F3oGT*) in flavonoid biosynthesis pathway. PAL, an enzyme in the phenylpropanoid pathway, is involved in physiological processes such as anthocyanin accumulation, lignification, flavonoid synthesis, and pathogen defenses. We find that the expression of *PAL* is significantly upregulated in the leaves of male sterile mutants and *PAL* has a significant positive correlation with rosmarinic acid and salvianolic acid B content. Therefore, the result confirms that *PAL* plays an important role in the synthesis of phenolic acid compounds in *S. miltiorrhiza*. Our results show that most of the genes in the male sterile mutants are significantly upregulated. Overexpression of *FLS* gene has effect on the accumulation of flavonoids [35]. Therefore, *FLS* gene could be a key enzyme encoding gene involved in the flavonoid biosynthesis. To validate the reliability of the transcriptome sequencing data, the sequences of seven important DEGs related to the phenylpropanoids pathway and the sucrose-starch metabolism pathway were analysed with RT-qPCR. The results of the RT-qPCR analysis exhibit a close similarity to the RNA-Seq results, as shown in Additional file 12: Figure S6.

## Discussion

Our results indicate that the male sterile mutants have reduced primary metabolism, but increased secondary metabolism in leaves. In order to understand the molecular mechanism for growth and metabolism in the male sterile mutants, we performed a comparative transcriptome and physiological analysis of the *S. miltiorrhiza* leaves between male fertile lines and male sterile lines. The height and biomass of male sterile lines are significantly less than that of male fertile lines, which might be responsible for significant change in the values of photosynthesis-related indicators, including leaf gas exchange (*A*, *E* and *g*_s_), chlorophyll fluorescence (F_v_, F_m_ and F_v_/F_m_) and chlorophyll content. Our findings are consistent with those of previous studies that conclude photosynthesis is reduced in male sterile mutants [36]. Additionally, we identify many DEGs related to photosynthesis, carbon fixation in photosynthetic organisms, sucrose-starch metabolism and phenylpropanoid metabolism pathway through RNA-seq analysis between male fertile lines and male sterile lines.

In Fig. 4c, we find structural abnormalities in the chloroplasts in male sterile mutants. Chloroplasts are highly specialized organelles in higher plants where photosynthesis including important metabolic pathways takes place [37]. Photosynthesis is the basic source of plant biomass yield. The difference in photosynthetic characteristics between varieties is often one of the direct causes of difference in growth. The analyses of leaf gas exchange characteristics, chlorophyll fluorescence indicators and photosynthesis pigments demonstrate that the photosynthetic capacity is significantly reduced in the MS mutants compared with the MF *S. miltiorrhiza* (Fig. 5). Consistent with this observation, the DEGs were significantly enriched in the photosynthesis pathway. In Fig. 5a, we find that all the values of the indicators except intercellular CO_2_ concentration (*C*_i_) suggested photosynthesis was reduced in the male sterile lines of *S. miltiorrhiza*. The research shows that net photosynthetic rate is 50% lower in male sterile mutants of *Nicotiana sylvestris* [36]. Other researchers have also reported significant reduction of photosynthetic rate in other male sterile plants [38, 39]. Our results show that the chloroplast structure is abnormal and the performance of photosynthesis is consistent with other research results [40]. The results also show that the net photosynthetic rate is positively correlated with the stomatal conductance, which agrees with the statistical analysis of other 14 species [41]. 90–95% plants᾽ dry matter of the above-ground parts comes from photosynthesis. Therefore, the most important thing to increase yield is to increase the utilization efficiency of light energy, the photosynthetic rate of plants, and the yield per unit area. In Fig. 5b and c, we find that the value of chlorophyll fluorescence indicators (F_m_, F_v_ and F_v_/F_m_) and chlorophyll content are lower in the male sterile lines of *S. miltiorrhiza*. It is found that the chlorophyll content is correlated to the photosynthetic activity. The morphological structures and physiological functions of the plants are correlated closely, which can be proved by the fact that the photosynthetic processes are correlated to the morphological characteristics of the photosynthetic organs of the leaves. Therefore, the results indicate that one of the causes for the stunted growth, decreased biomass and decreased primary metabolism in the male sterile *S. miltiorrhiza* mutants could be due to the abnormal chloroplast structure, which significantly reduces the plant’s physiological activities and performances such as gas exchange and chlorophyll fluorescence and chlorophyll content.

In this study, we find that the chloroplast structure in the male sterile mutants of *S. miltiorrhiza* is not fully developed. The abnormal development of chloroplast structure in male sterile mutants might be related to DEGs involved in photosynthesis-related processes. In Fig. 6, a detailed analysis of the RNA-seq data indicates that 1, 1, 1 and 6 genes, which are usually related to PSI, PSII, photosynthetic electron transport and carbon fixation in photosynthetic organisms, respectively, are significantly differentially expressed between two lines. The most of these genes are downregulated in male sterile lines of *S. miltiorrhiza*. These results are consistent with the phenotypic changes including photosynthesis rate, chlorophyll fluorescence, and chlorophyll content (Fig. 5). Photosynthesis is considered a major determinant of yield [42]. Ribulose 1,5-bisphosphate carboxylase/oxygenase (Rubisco) activities are associated with photosynthetic rate-limiting steps [43, 44]. A comprehensive analysis of our RNA-seq data reveals that the expression level of *rbcS* is sharply decreased in male sterile lines (Fig. 6). FBA is a key enzyme in plants that is involved in gluconeogenesis, glycolysis, and the Calvin cycle. FBA genes play significant roles in regulating growth and development [45]. Reduction in sucrose content caused by downregulation of *FBP* results in tiller outgrowth cessation in rice mutants [46]. Thus, the abnormal development of chloroplast structure in male sterile mutants might be related to the downregulated enzymes (i.g., *rbcS*, *FBA* and *FBP*) in photosynthesis-related processes.

In Fig. 7, the DEGs are mainly involved in the degradation process of sucrose and starch. Therefore, we select the genes as important genes which encode enzymes *SUS3*, *CINV2* and *DPE1* that are significantly upregulated in the sucrose-starch metabolism pathway. In most of higher plants, sucrose is a main sugar that moves from leaves through phloem to other tissues. Sucrose then needs to be hydrolyzed into hexoses by either INV or SUS [47], and our results just confirm this conclusion. Researchers have found that lack of *DPE1* in plants causes slightly more accumulation of starch in the leaves than in the control plants [48], which agrees with our results. Therefore, the results suggest that one of the reasons for the decrease in sucrose and starch in male sterile mutants of *S. miltiorrhiza* might be due to fast degradation of starch, which is catalyzed by DPE1 and fast degradation of sucrose, which is catalyzed by SUS3 and CINV2.

In Fig. 8, we find that there is a significant upregulation of *PAL* in *S. miltiorrhiza* leaves of the male sterile mutants. Researchers have found that *PAL* has a significant positive correlation with rosmarinic acid and salvianolic acid B content, and our results match this conclusion [49]. Therefore, *PAL* plays an important role in the synthesis of phenolic acid compounds in *S. miltiorrhiza*. In this study, it is also found that male sterile mutants have higher relative content of total flavonoid and total phenolic (Fig. 1b). Flavonoids and polyphenols are common secondary metabolites in higher plants and play an important role in diverse activities. For example, they can protect plants from pathogenic infection, provide flowers with pigment to attract pollinators, and reduce the risk of oxidative damage to human health [50–52]. In Fig. 8, we select the *FLS* gene*,* whose expression is significantly upregulated, as an important gene which is involved in the flavonoid biosynthesis pathway. The expression of *FLS* gene requires activation of the MYB transcription factor [53]. Upregulated expression of *FLS* gene affects the accumulation of flavonoids [35]. Therefore, *FLS* gene could be a key enzyme encoding gene affecting the synthesis of flavonoids. In addition, our research on the genes related to the metabolic pathways could provide useful information for metabolic engineering strategies to control the overall metabolic flux of potent antioxidants that have beneficial implications for human health.

## Conclusions

In conclusion, our results show that the male sterile lines of *S. miltiorrhiza* have lower primary metabolism and more active secondary metabolism in leaves. In order to understand the molecular mechanism of plant growth and metabolic yield in the male sterile mutants, we performed the transcriptome and physiological analysis of the *S. miltiorrhiza* leaves of male fertile lines and male sterile lines. The differences in physiological processes and activities between the two line lie with the different development of their chloroplast structures. The abnormal chloroplast structure of male fertile leaves decreases the leaf gas exchange (*A*, *E* and *g*_s_), chlorophyll fluorescence (F_v_, F_m_ and F_v_/F_m_) and chlorophyll content. The results confirm that DPE1 catalyzes the degradation of starch, and SUS3 and CINV2 catalyzes the degradation of sucrose in *S. miltiorrhiza*. At the same time, the results suggest that *PAL* plays a vital role in the biosynthesis of rosmarinic acid and salvianolic acid B and *FLS* is essential for the biosynthesis of flavonoids. Taken together, our results from the physiological and transcriptomic analysis reveal underlying mechanisms of plant growth and metabolic yield in the male sterile mutants.

## Supplementary information

**Additional file 1: Figure S1.** The phenotype of flower and stem and the determination of active ingredients content in flowers and roots between MF and MS in *S. miltiorrhiza*. (**A**) The phenotypes of flower; (**B**) The phenotypes of stem; (**C**) The content of salvianolic acid B and rosmarinic acid in flowers; (**D**) The content of salvianolic acid B and rosmarinic acid in roots between MF and MS in *S. miltiorrhiza* and (**E**) The content of the tanshinones (including tanshinone I, tanshinone II-A, dihydrotanshinone and cryptotanshinone) in roots between MF and MS in *S. miltiorrhiza*.

**Additional file 2: Table S1.** Primer sequences of DEGs between MF and MS in *S. miltiorrhiza* leaves.

**Additional file 3: Table S2.** The base information before and after filtration.

**Additional file 4: Table S3.** The reads information before and after filtering.

**Additional file 5: Table S4.** The statistics of ribosomal alignment results.

**Additional file 6: Figure S2.** The distribution of genes coverage. The three biological replicates of male fertility are F1, F2 and F3, and the three biological replicates of male sterility are S1, S2 and S3. In the figure, A, B, and C represent F1, F2, and F3, respectively. And D, E, and F represent S1, S2, and S3, respectively.

**Additional file 7: Figure S3.** The classification of raw reads. The three biological replicates of male fertility are F1, F2 and F3, and the three biological replicates of male sterility are S1, S2 and S3.

**Additional file 8: Table S5.** Pathway enrichment of DEGs between MF and MS in *S. miltiorrhiza* leaves.

**Additional file 9: Figure S4.** KEGG classified into five largest pathways between near-isogenic male fertile and male sterile lines in *S. miltiorrhiza* leaves.

**Additional file 10: Excel S1.** Mapping files of nucleic acid sequences in *S. miltiorrhiza*.

**Additional file 11: Figure S5.** Partial phenylpropanoids pathway between MF and MS in *S. miltiorrhiza* leaves. The figure showed the result of MS compared against MF. Blue represents downregulated and red represents up-regulated. PAL: Phenylalanine ammonialyase; C4H: Cinnamate 4-hydroxylase; 4CL: 4-coumarate-CoA ligase; CCR: Cinnamoyl-CoA reductase; COMT: Caffeic acid O-methyltransferase; F5H: Ferulic acid-5-hydroxylase.

**Additional file 12: Figure S6.** Expression verification of seven candidates from MF and MS in *S. miltiorrhiza* leaves. β-actin was used as an internal control. Each gene has three biological replicates and three technical replicates.

## Data Availability

The datasets supporting the conclusions of this article are included with in the article and its additional files.

## Abbreviations

DEGs: Differentially expressed genes
FC: fold change
FDR: False discovery rate
FPKM: Fragments Per Kilobase of transcript per Million mapped reads
GO: Gene Ontology
KEGG: Kyoto Encyclopedia of Genes and Genomes
MF: Near-isogenic male fertile lines
MS: Near-isogenic male sterile lines
N50: Covering 50% of all the nucleotide sequences of the largest unigene length
Q30 percentage: Percentage of bases with sequencing error rate lower than 1‰
RNA-Seq: RNA sequencing
RT-qPCR: Real-time quantitative polymerase chain reaction
TEM: Transmission electron microscopy

## References

[1] Mishra S, Kumari V (2018). A review on male sterility-concepts and utilization in vegetable crops. Int J Curr Microbiol App Sci.

[2] Toppino L, Kooiker M, Lindner M, Dreni L, Rotino GL, Kater MM (2011). Reversible male sterility in eggplant (*Solanum melongena* L.) by artificial microRNA-mediated silencing of general transcription factor genes. Plant Biotechnol J.

[3] Xu C, Liu Z, Zhang L, Zhao C, Yuan S, Zhang F (2013). Organization of actin cytoskeleton during meiosis I in a wheat thermo-sensitive genic male sterile line. Protoplasma.

[4] Singh SP, Srivastava R, Kumar J (2014). Male sterility systems in wheat and opportunities for hybrid wheat development. Acta Physiol Plant.

[5] Zhang H, Xu C, He Y, Zong J, Yang X, Si H (2013). Mutants in CSA creates a new photoperiod-sensitive genic male sterile line applicable for hybrid rice seed yield. Proc Natl Acad Sci U S A.

[6] Li Z, Cheng Y, Cui J, Zhang P, Zhao H, Hu S (2015). Comparative transcriptome analysis reveals carbohydrate and lipid metabolism blocks in *Brassica napus* L male sterility induced by the chemical hybridization agent monosulfuron ester sodium. BMC Genomics.

[7] Shu Z, Wang Z, Mu X, Liang Z, Guo H (2012). A dominant gene for male sterility in *Salvia miltiorrhiza* Bunge. PLoS One.

[8] Haddad IVN, Ribeiro de Santiago-Fernandes LD, Machado SR (2018). Autophagy is associated with male sterility in pistillate flowers of *Maytenus obtusifolia* (Celastraceae). Aus J Botany.

[9] Wan X, Wu S, Li Z, Dong Z, An X, Ma B, Tian Y, Li J (2019). Maize genic male-sterility genes and their applications in hybrid breeding: Progress and perspectives. Mol Plant.

[10] Tang H, Xie Y, Liu YG, Chen L (2017). Advances in understanding the molecular mechanisms of cytoplasmic male sterility and restoration in rice. Plant Reprod.

[11] Engelke T, Hirsche J, Roitsch T (2010). Anther-specific carbohydrate supply and restoration of metabolically engineered male sterility. J Exp Bot.

[12] Horn R, Gupta KJ, Colombo N (2014). Mitochondrion role in molecular basis of cytoplasmic male sterility. Mitochondrion.

[13] Hu J, Huang W, Huang Q, Qin X, Yu C, Wang L, Li S, Zhu R, Zhu Y (2014). Mitochondria and cytoplasmic male sterility in plants. Mitochondrion.

[14] Roth GA, Johnson C, Abajobir A, Abd-Allah F, Abera SF, Abyu G (2017). Global, regional, and National Burden of cardiovascular diseases for 10 causes, 1990 to 2015. J Am Coll Cardiol.

[15] Townsend N, Wilson L, Bhatnagar P, Wickramasinghe K, Rayner M, Nichols M (2016). Cardiovascular disease in Europe: epidemiological update 2016. Eur Heart J.

[16] Xu H, Song J, Luo H, Zhang Y, Li Q, Zhu Y (2016). Analysis of the genome sequence of the medicinal plant *Salvia miltiorrhiza*. Mol Plant.

[17] Pei T, Ma P, Ding K, Liu S, Jia Y, Ru M, Dong J, Liang Z (2018). SmJAZ8 acts as a core repressor regulating JA-induced biosynthesis of salvianolic acids and tanshinones in *Salvia miltiorrhiza* hairy roots. J Exp Bot.

[18] Zhang G, Tian Y, Zhang J, Shu L, Yang S, Wang W, Sheng J, Dong Y, Chen W (2015). Hybrid de novo genome assembly of the Chinese herbal plant danshen (*Salvia miltiorrhiza* Bunge). Gigascience.

[19] Song J, Ji Y, Xu K, Wang Z (2012). An integrated analysis of the rosmarinic acid-biosynthetic genes to uncover the regulation of rosmarinic acid pathway in *Salvia miltiorrhiza*. Acta Physiol Plant.

[20] Zhang Y, Guo L, Shu Z, Sun Y, Chen Y, Liang Z, Guo H (2013). Identification of amplified fragment length polymorphism (AFLP) markers tightly associated with drought stress gene in male sterile and fertile *Salvia miltiorrhiza* Bunge. Int J Mol Sci.

[21] Ainsworth EA, Gillespie KM (2007). Estimation of total phenolic content and other oxidation substrates in plant tissues using Folin-Ciocalteu reagent. Nat Protoc.

[22] Zilani MNH, Sultana NA, Bakshi MK, Shampa IJ, Sumi SJ, Islam O (2018). Bioactivities of leaf and root extract of *Ceriscoids turgida* (Roxb.). Orient Pharm Exp Med.

[23] Liang ZS, Yang DF, Liang X, Zhang YJ, Liu Y, Liu FH (2012). Roles of reactive oxygen species in methyl jasmonate and nitric oxide-induced tanshinone yield in *Salvia miltiorrhiza* hairy roots. Plant Cell Rep.

[24] Langmead B, Salzberg SL (2012). Fast gapped-read alignment with bowtie 2. Nat Methods.

[25] Li B, Dewey CN (2011). RSEM: accurate transcript quantification from RNA-Seq data with or without a reference genome. BMC Bioinformatics.

[26] Love MI, Huber W, Anders S (2014). Moderated estimation of fold change and dispersion for RNA-seq data with DESeq2. Genome Biol.

[27] Kanehisa M, Araki M, Goto S, Hattori M, Hirakawa M, Itoh M (2008). KEGG for linking genomes to life and the environment. Nucleic Acids Res.

[28] Chen S, Bai Y, Zhang L, Han X (2005). Comparing physiological responses of two dominant grass species to nitrogen addition in Xilin River basin of China. Environ Exp Bot.

[29] Matkowski A, Zielinska S, Oszmianski J, Lamer-Zarawska E (2008). Antioxidant activity of extracts from leaves and roots of *Salvia miltiorrhiza* Bunge, S. przewalskii maxim., and *S. verticillata* L. Bioresour Technol.

[30] Ali M, Abbasi BH (2013). Ihsan ul h. yield of commercially important secondary metabolites and antioxidant activity in cell suspension cultures of *Artemisia absinthium* L. Ind Crop Prod.

[31] Athar HR, Zafar ZU, Ashraf M (2015). Glycinebetaine improved photosynthesis in canola under salt stress: evaluation of chlorophyll fluorescence parameters as potential indicators. J Agron Crop Sci.

[32] Chandran AKN, Lee GS, Yoo YH, Yoon UH, Ahn BO, Yun DW, et al. Functional classification of rice flanking sequence tagged genes using MapMan terms and global understanding on metabolic and regulatory pathways affected by *dxr* mutants having defects in light response. Rice (N Y). 2016;9(1):–17. 10.1186/s12284-016-0089-2 PMC4830809 27076183

[33] Goffard N, Weiller G (2006). Extending MapMan: application to legume genome arrays. Bioinformatics..

[34] Hao C, Xia Z, Fan R, Tan L, Hu L, Wu B, Wu H (2016). De novo transcriptome sequencing of black pepper (*Piper nigrum* L.) and an analysis of genes involved in phenylpropanoid metabolism in response to Phytophthora capsici. BMC Genomics.

[35] Nguyen NH, Kim JH, Kwon J, Jeong CY, Lee W, Lee D, Hong S-W, Lee H (2016). Characterization of *Arabidopsis thaliana* FLAVONOL SYNTHASE 1 (*FLS1*) -overexpression plants in response to abiotic stress. Plant Physiol Biochem.

[36] Sabar M, De Paepe R, de Kouchkovsky Y (2000). Complex I impairment, respiratory compensations, and photosynthetic decrease in nuclear and mitochondrial male sterile mutants of *Nicotiana sylvestris*. Plant Physiol.

[37] Cortleven A, Schmulling T (2015). Regulation of chloroplast development and function by cytokinin. J Exp Bot.

[38] Lian JL, Ren LS, Zhang C, Yu CY, Huang Z, Xu AX, Dong JG (2019). How exposure to ALS-inhibiting gametocide tribenuron-methyl induces male sterility in rapeseed. BMC Plant Biol.

[39] Xie F, Yuan JL, Li YX, Wang CJ, Tang HY, Xia JH, Yang QY, Wan ZJ (2018). Transcriptome Analysis Reveals Candidate Genes Associated with Leaf Etiolation of a Cytoplasmic Male Sterility Line in Chinese Cabbage (*Brassica Rapa* L. ssp. Pekinensis). Int J Mol Sci.

[40] Lv Y, Shao G, Qiu J, Jiao G, Sheng Z, Xie L (2017). White leaf and panicle 2, encoding a PEP-associated protein, is required for chloroplast biogenesis under heat stress in rice. J Exp Bot.

[41] Gago J, Daloso Dde M, Figueroa CM, Flexas J, Fernie AR, Nikoloski Z (2016). Relationships of leaf net photosynthesis, Stomatal conductance, and mesophyll conductance to primary metabolism: a multispecies meta-analysis approach. Plant Physiol.

[42] Niinemets U, Berry JA, von Caemmerer S, Ort DR, Parry MA, Poorter H (2017). Photosynthesis: ancient, essential, complex, diverse ... and in need of improvement in a changing world. New Phytol.

[43] Hermida-Carrera C, Kapralov MV, Galmes J (2016). Rubisco catalytic properties and temperature response in crops. Plant Physiol.

[44] Orr DJ, Alcantara A, Kapralov MV, Andralojc PJ, Carmo-Silva E, Parry MA (2016). Surveying Rubisco diversity and temperature response to improve crop photosynthetic efficiency. Plant Physiol.

[45] Lv GY, Guo XG, Xie LP, Xie CG, Zhang XH, Yang Y (2017). Molecular characterization, gene evolution, and expression analysis of the Fructose-1, 6-bisphosphate Aldolase (*FBA*) gene family in wheat (*Triticum aestivum* L.). front. Plant Sci.

[46] Ohashi M, Ishiyama K, Kusano M, Fukushima A, Kojima S, Hayakawa T, Yamaya T (2018). Reduction in sucrose contents by downregulation of fructose-1,6-bisphosphatase 2 causes tiller outgrowth cessation in rice mutants lacking glutamine synthetase1;2. Rice (N Y).

[47] Ruan YL (2012). Signaling role of sucrose metabolism in development. Mol Plant.

[48] Lutken H, Lloyd JR, Glaring MA, Baunsgaard L, Laursen KH, Haldrup A, Kossmann J, Blennow A (2010). Repression of both isoforms of disproportionating enzyme leads to higher malto-oligosaccharide content and reduced growth in potato. Planta..

[49] Zhang X, Liu CJ (2015). Multifaceted regulations of gateway enzyme phenylalanine ammonia-lyase in the biosynthesis of phenylpropanoids. Mol Plant.

[50] Panche AN, Diwan AD, Chandra SR (2016). Flavonoids: an overview. J Nutr Sci.

[51] George VC, Dellaire G, Rupasinghe HPV (2017). Plant flavonoids in cancer chemoprevention: role in genome stability. J Nutr Biochem.

[52] Kallscheuer N, Vogt M, Bott M, Marienhagen J (2017). Functional expression of plant-derived O-methyltransferase, flavanone 3-hydroxylase, and flavonol synthase in Corynebacterium glutamicum for yield of pterostilbene, kaempferol, and quercetin. J Biotechnol.

[53] Matsui K, Oshima Y, Mitsuda N, Sakamoto S, Nishiba Y, Walker AR (2018). Buckwheat R2R3 MYB transcription factor FeMYBF1 regulates flavonol biosynthesis. Plant Sci.

