# Supplementary figures and images for: Physiological and transcriptome analysis reveal molecular mechanism in Salvia miltiorrhiza leaves of near-isogenic male fertile lines and male sterile lines
Source: BMC Genomics. 2019 Oct 26;20:780. doi: 10.1186/s12864-019-6173-4 (PMC6815445; doi:10.1186/s12864-019-6173-4)

**A**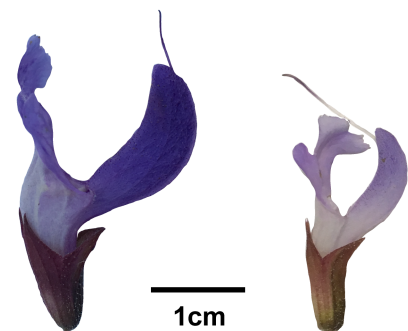**B**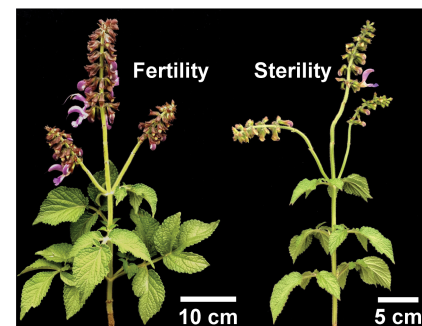**C**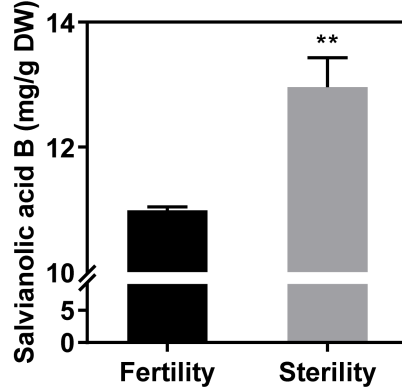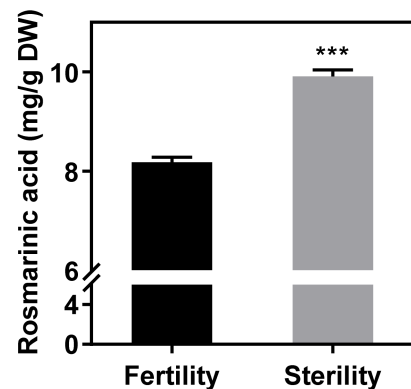**D**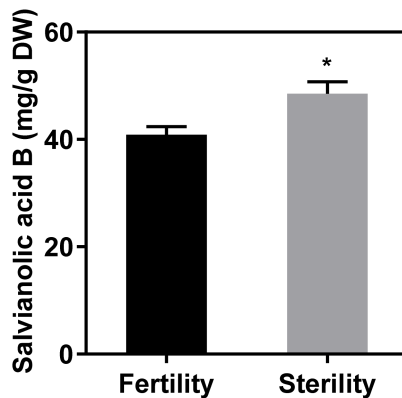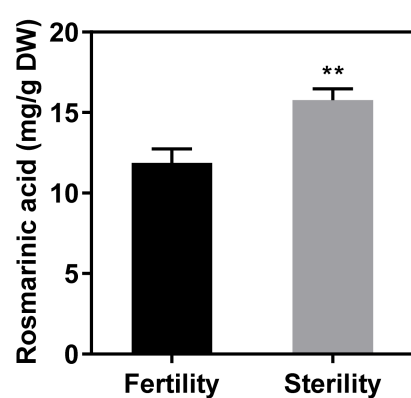**E**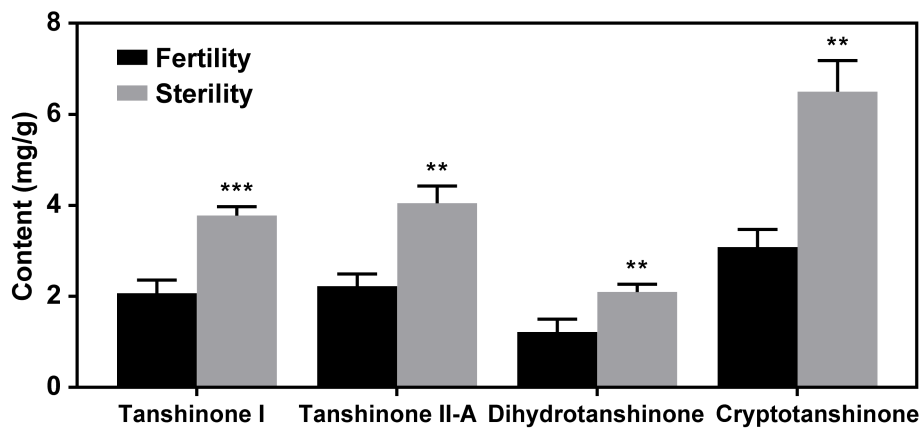

Supplement: Supplementary file 1 — Additional file 1: Figure S1. The phenotype of flower and stem and the determination of active ingredients content in flowers and roots between MF and MS in S. miltiorrhiza. (A) The phenotypes of flower; (B) The phenotypes of stem; (C) The content of salvianolic acid B and rosmarinic acid in flowers; (D) The content of salvianolic acid B and rosmarinic acid in roots between MF and MS in S. miltiorrhiza and (E) The content of the tanshinones (including tanshinone I, tanshinone II-A, dihydrotanshinone and cryptotanshinone) in roots between MF and MS in S. miltiorrhiza. [file 12864_2019_6173_MOESM1_ESM.pdf]

**A**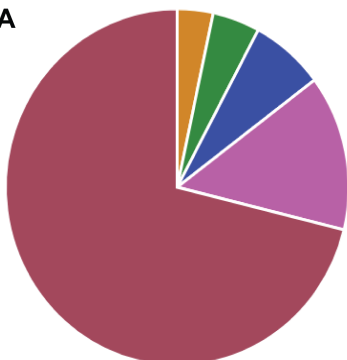**B**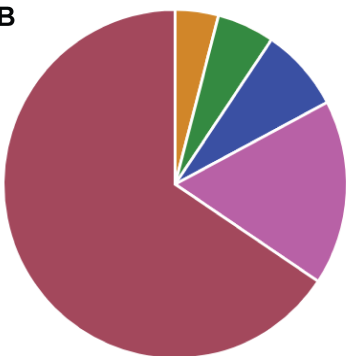**C**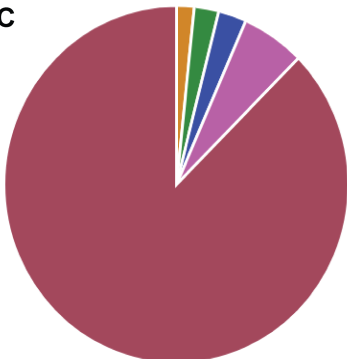**D**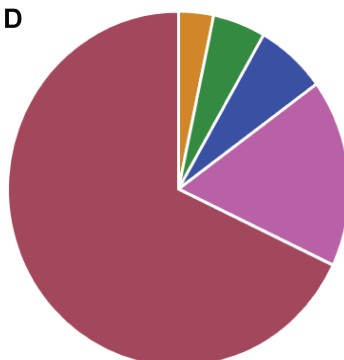**E**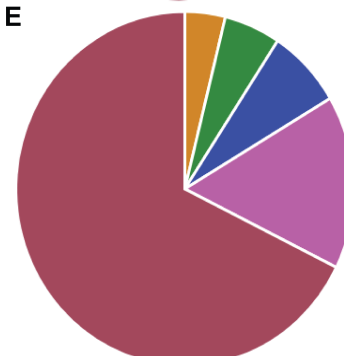**F**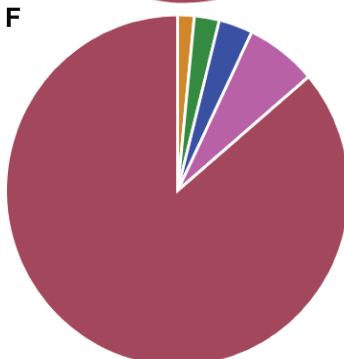

Supplement: Supplementary file 6 — Additional file 6: Figure S2. The distribution of genes coverage. The three biological replicates of male fertility are F1, F2 and F3, and the three biological replicates of male sterility are S1, S2 and S3. In the figure, A, B, and C represent F1, F2, and F3, respectively. And D, E, and F represent S1, S2, and S3, respectively. [file 12864_2019_6173_MOESM6_ESM.pdf]

**Classification of Raw Reads**

**Sample**

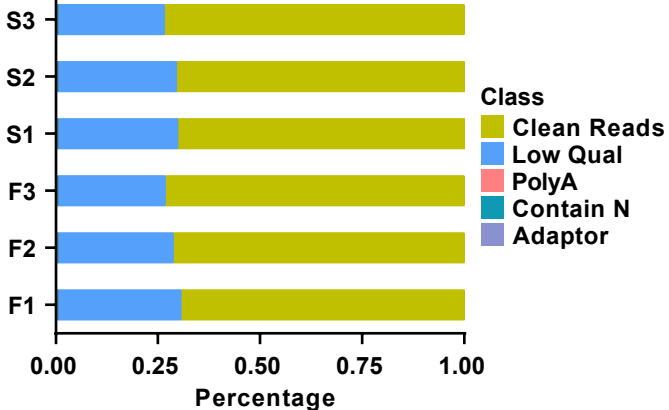

Supplement: Supplementary file 7 — Additional file 7: Figure S3. The classification of raw reads. The three biological replicates of male fertility are F1, F2 and F3, and the three biological replicates of male sterility are S1, S2 and S3. [file 12864_2019_6173_MOESM7_ESM.pdf]

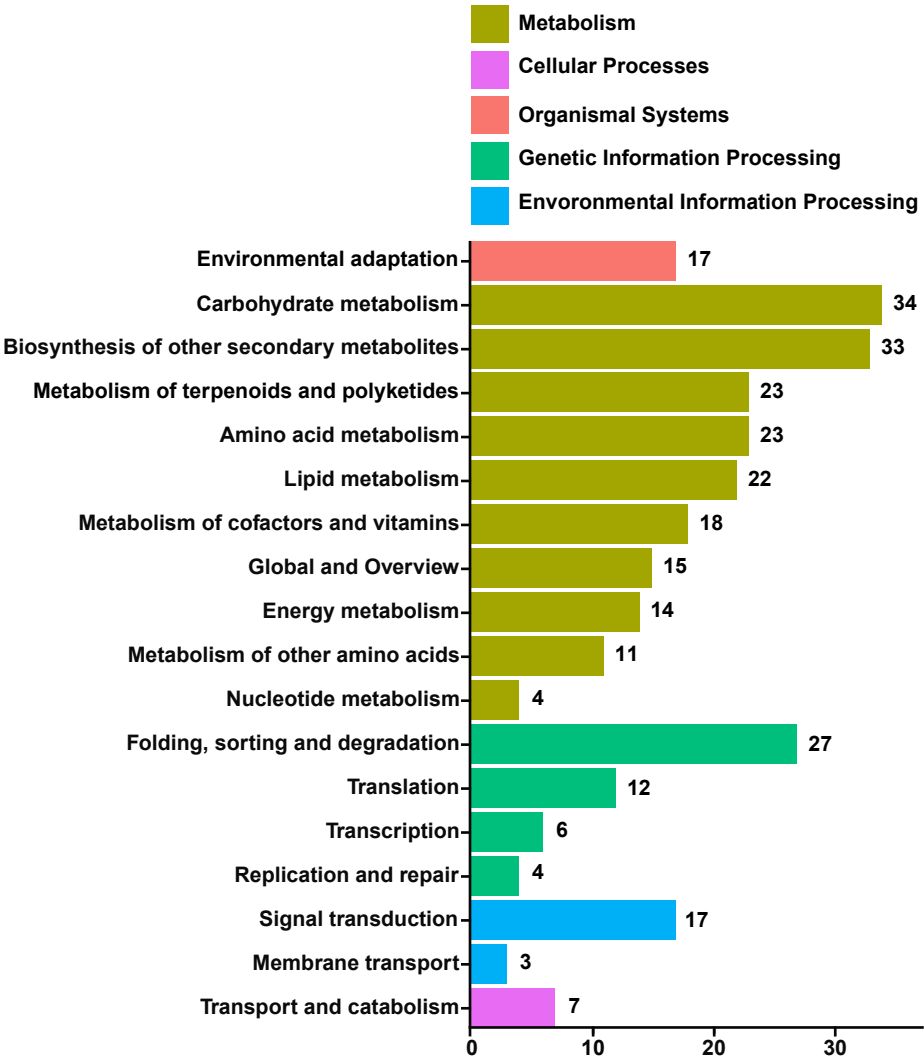

Supplement: Supplementary file 9 — Additional file 9: Figure S4. KEGG classified into five largest pathways between near-isogenic male fertile and male sterile lines in S. miltiorrhiza leaves. [file 12864_2019_6173_MOESM9_ESM.pdf]

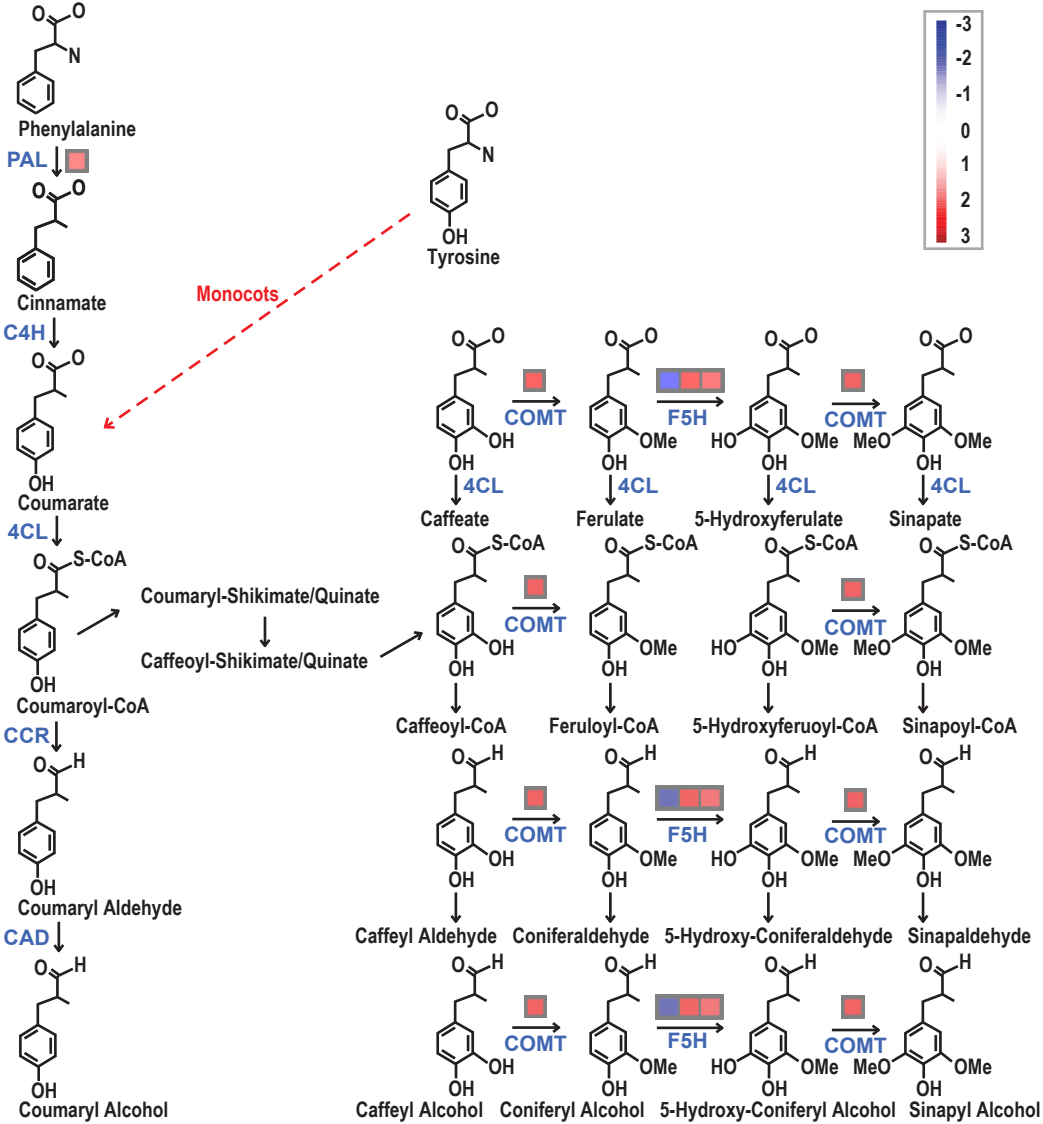

Supplement: Supplementary file 11 — Additional file 11: Figure S5. Partial phenylpropanoids pathway between MF and MS in S. miltiorrhiza leaves. The figure showed the result of MS compared against MF. Blue represents downregulated and red represents up-regulated. PAL: Phenylalanine ammonialyase; C4H: Cinnamate 4-hydroxylase; 4CL: 4-coumarate-CoA ligase; CCR: Cinnamoyl-CoA reductase; COMT: Caffeic acid O-methyltransferase; F5H: Ferulic acid-5-hydroxylase. [file 12864_2019_6173_MOESM11_ESM.pdf]

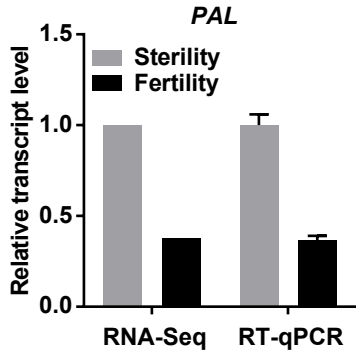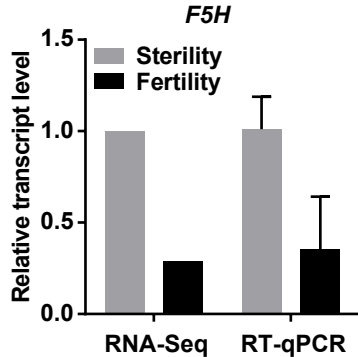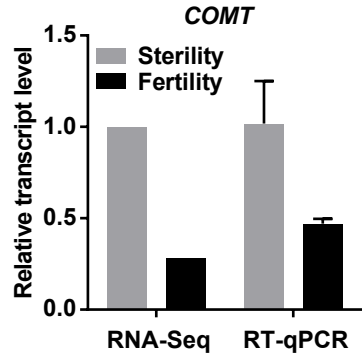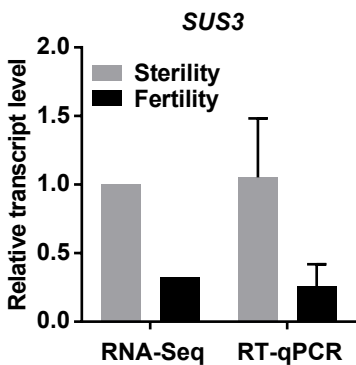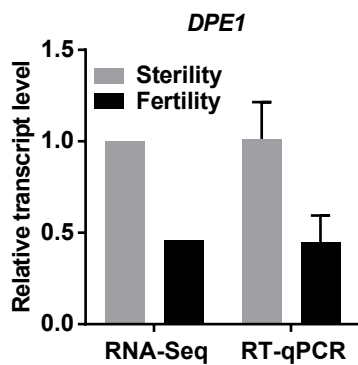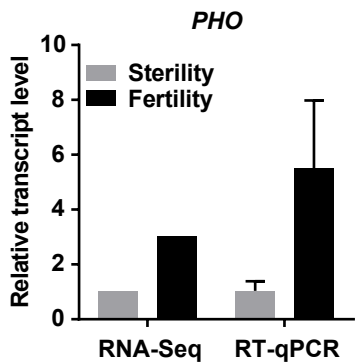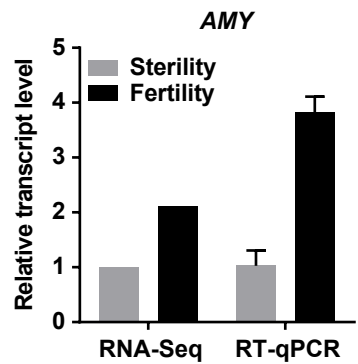

Supplement: Supplementary file 12 — Additional file 12: Figure S6. Expression verification of seven candidates from MF and MS in S. miltiorrhiza leaves. β-actin was used as an internal control. Each gene has three biological replicates and three technical replicates. [file 12864_2019_6173_MOESM12_ESM.pdf]
