# Supplementary material for: Physiological and transcriptome analysis reveal molecular mechanism in Salvia miltiorrhiza leaves of near-isogenic male fertile lines and male sterile lines
Source: BMC Genomics. 2019 Oct 26;20:780. doi: 10.1186/s12864-019-6173-4 (PMC6815445; doi:10.1186/s12864-019-6173-4)
Supplement: Supplementary file 2 — Additional file 2: Table S1. Primer sequences of DEGs between MF and MS in S. miltiorrhiza leaves. [file 12864_2019_6173_MOESM2_ESM.doc]

**Table S1** Primer sequences of DEGs between near-isogenic male fertile lines and male sterile lines in *S. miltiorrhiza* leaves

| Gene ID | Gene name | Forward primer (5᾽- 3᾽) | Tm | Reverse primer (5᾽- 3᾽) | Tm |
| --- | --- | --- | --- | --- | --- |
| evm.model.scaffold1643.3 | *PAL* | GGACCACCTCACCCACAAGC | 60.06 | CCCTCTGCGCCTCCTTGATG | 59.99 |
| evm.model.scaffold6088.3 | *COMT* | CCGGTTCCGGCGTCTTTCTT | 60.13 | AGGCGGAGGATCCGGTCAAT | 60.20 |
| evm.model.C216999.5 | *F5H* | GACGGCATCATCCACGAGCA | 59.99 | ACTCCAGCTCTCCGCTTTCCT | 60.06 |
| evm.model.C213815.1 | *SUS3* | CAGAGCCCGTAACGGTGAGC | 60.27 | GACCGCCATGGCAAGTAGCA | 60.20 |
| evm.model.scaffold4902.1 | *PHO* | TACCACGCCACCGCTCAAAG | 60.13 | CAGCAAGGCTCTGCCCTGAA | 59.78 |
| evm.model.scaffold1944.2 | *AMY* | GGGATGGTGGCCTTCTCGTG | 59.92 | ACAGGAGTCCCAGGATGCGT | 60.06 |
| evm.model.scaffold4264.1 | *DPE1* | CCTTCCTCCCACGCCCAATC | 59.92 | GCTTCGGCATCCAGCTCTCA | 59.64 |
|  | *β-actin* | GGTGCCCTGAGGTCCTGTT | 59.20 | AGGAACCACCGATCCAGACA | 59.70 |
