# Supplementary material for: Physiological and transcriptome analysis reveal molecular mechanism in Salvia miltiorrhiza leaves of near-isogenic male fertile lines and male sterile lines
Source: BMC Genomics. 2019 Oct 26;20:780. doi: 10.1186/s12864-019-6173-4 (PMC6815445; doi:10.1186/s12864-019-6173-4)
Supplement: Supplementary file 3 — Additional file 3: Table S2. The base information before and after filtration. [file 12864_2019_6173_MOESM3_ESM.doc]

**Table S2** The base information before and after filtration

| Sample | Bases | Reads | Length (Max/Mean/Min) | Bases (%) | Reads (%) | Length (Max/Mean/Min) |
| --- | --- | --- | --- | --- | --- | --- |
| F1 | 1890000000 | 15000000 | 126/126/126 | 1294326900 (68.48%) | 10432766 (69.55%) | 126/124/50 |
| F2 | 1890000000 | 15000000 | 126/126/126 | 1320757930 (69.88%) | 10710398 (71.40%) | 126/123/50 |
| F3 | 7674026472 | 60904972 | 126/126/126 | 5533347682 (72.10%) | 44701706 (73.40%) | 126/123/50 |
| S1 | 1890000000 | 15000000 | 126/126/126 | 1304759958 (69.03%) | 10549154 (70.33%) | 126/123/50 |
| S2 | 1890000000 | 15000000 | 126/126/126 | 1308057108 (69.21%) | 10596970 (70.65%) | 126/123/50 |
| S3 | 8035850340 | 63776590 | 126/126/126 | 5806807250 (72.26%) | 46953660 (73.62%) | 126/123/50 |

Note: The three biological replicates of male fertility are F1, F2 and F3, and the three biological replicates of male sterility are S1, S2 and S3.
