# Supplementary material for: Physiological and transcriptome analysis reveal molecular mechanism in Salvia miltiorrhiza leaves of near-isogenic male fertile lines and male sterile lines
Source: BMC Genomics. 2019 Oct 26;20:780. doi: 10.1186/s12864-019-6173-4 (PMC6815445; doi:10.1186/s12864-019-6173-4)
Supplement: Supplementary file 4 — Additional file 4: Table S3. The reads information before and after filtering. [file 12864_2019_6173_MOESM4_ESM.doc]

**Table S3** The reads information before and after filtering

| Sample | Q20 | Q30 | GC | N | Q20 | Q30 | GC | N |
| --- | --- | --- | --- | --- | --- | --- | --- | --- |
| F1 | 92.19% | 86.22% | 48.35% | 0.26% | 97.94% | 94.83% | 47.99% | 0.00% |
| F2 | 92.59% | 86.79% | 49.83% | 0.27% | 98.08% | 95.11% | 49.37% | 0.00% |
| F3 | 93.16% | 87.87% | 48.31% | 0.24% | 98.24% | 95.56% | 47.91% | 0.00% |
| S1 | 92.33% | 86.45% | 48.67% | 0.26% | 98.00% | 94.96% | 48.27% | 0.00% |
| S2 | 92.39% | 86.53% | 48.67% | 0.26% | 97.99% | 94.93% | 48.27% | 0.00% |
| S3 | 93.18% | 87.88% | 48.60% | 0.25% | 98.25% | 95.57% | 48.24% | 0.00% |

Note: The three biological replicates of male fertility are F1, F2 and F3, and the three biological replicates of male sterility are S1, S2 and S3.
