# Supplementary material for: Physiological and transcriptome analysis reveal molecular mechanism in Salvia miltiorrhiza leaves of near-isogenic male fertile lines and male sterile lines
Source: BMC Genomics. 2019 Oct 26;20:780. doi: 10.1186/s12864-019-6173-4 (PMC6815445; doi:10.1186/s12864-019-6173-4)
Supplement: Supplementary file 5 — Additional file 5: Table S4. The statistics of ribosomal alignment results. [file 12864_2019_6173_MOESM5_ESM.doc]

**Table S4** The statistics of ribosomal alignment results

| Sample | All Reads Num | Mapped Reads | Unmapped Reads |
| --- | --- | --- | --- |
| F1 | 10432766 | 212784 (2.04%) | 10219982 (97.96%) |
| F2 | 10710398 | 244928 (2.29%) | 10465470 (97.71%) |
| F3 | 44701706 | 247614 (0.55%) | 44454092 (99.45%) |
| S1 | 10549154 | 510822 (4.84%) | 10038332 (95.16%) |
| S2 | 10596970 | 90336 (0.85%) | 10506634 (99.15%) |
| S3 | 46953660 | 898016 (1.91%) | 46055644 (98.09%) |

Note: The three biological replicates of male fertility are F1, F2 and F3, and the three biological replicates of male sterility are S1, S2 and S3.
