# Supplementary material for: Physiological and transcriptome analysis reveal molecular mechanism in Salvia miltiorrhiza leaves of near-isogenic male fertile lines and male sterile lines
Source: BMC Genomics. 2019 Oct 26;20:780. doi: 10.1186/s12864-019-6173-4 (PMC6815445; doi:10.1186/s12864-019-6173-4)
Supplement: Supplementary file 8 — Additional file 8: Table S5. Pathway enrichment of DEGs between MF and MS in S. miltiorrhiza leaves. [file 12864_2019_6173_MOESM8_ESM.doc]

**Table S5** Pathway enrichment of DEGs between near-isogenic male fertile and male sterile lines in *S. miltiorrhiza* leaves

| # | Pathway | DEGs genes with pathway annotation (231) | All genes with pathway annotation (5987) | Pvalue | Qvalue | Pathway ID |
| --- | --- | --- | --- | --- | --- | --- |
| 1 | Sesquiterpenoid and triterpenoid biosynthesis | 9 (3.9%) | 27 (0.45%) | 0 | 0.000037 | ko00909 |
| 2 | Isoquinoline alkaloid biosynthesis | 9 (3.9%) | 57 (0.95%) | 0.000289 | 0.010388 | ko00950 |
| 3 | Taurine and hypotaurine metabolism | 5 (2.16%) | 17 (0.28%) | 0.000346 | 0.010388 | ko00430 |
| 4 | Glutathione metabolism | 11 (4.76%) | 96 (1.6%) | 0.001083 | 0.024369 | ko00480 |
| 5 | Tyrosine metabolism | 9 (3.9%) | 83 (1.39%) | 0.004429 | 0.079454 | ko00350 |
| 6 | Arachidonic acid metabolism | 4 (1.73%) | 19 (0.32%) | 0.005297 | 0.079454 | ko00590 |
| 7 | Protein processing in endoplasmic reticulum | 19 (8.23%) | 265 (4.43%) | 0.006434 | 0.082729 | ko04141 |
| 8 | Flavonoid biosynthesis | 7 (3.03%) | 63 (1.05%) | 0.010217 | 0.114945 | ko00941 |
| 9 | Cyanoamino acid metabolism | 6 (2.6%) | 54 (0.9%) | 0.016987 | 0.169875 | ko00460 |
| 10 | Phenylpropanoid biosynthesis | 14 (6.06%) | 198 (3.31%) | 0.02033 | 0.182973 | ko00940 |
| 11 | Anthocyanin biosynthesis | 2 (0.87%) | 9 (0.15%) | 0.044617 | 0.36505 | ko00942 |
| 12 | Carbon fixation in photosynthetic organisms | 7 (3.03%) | 95 (1.59%) | 0.073233 | 0.497451 | ko00710 |
| 13 | Cutin, suberine and wax biosynthesis | 4 (1.73%) | 42 (0.7%) | 0.077135 | 0.497451 | ko00073 |
| 14 | Brassinosteroid biosynthesis | 2 (0.87%) | 13 (0.22%) | 0.087428 | 0.497451 | ko00905 |
| 15 | Plant-pathogen interaction | 13 (5.63%) | 223 (3.72%) | 0.089091 | 0.497451 | ko04626 |
| 16 | Amino sugar and nucleotide sugar metabolism | 11 (4.76%) | 183 (3.06%) | 0.095349 | 0.497451 | ko00520 |
| 17 | Nicotinate and nicotinamide metabolism | 3 (1.3%) | 29 (0.48%) | 0.09936 | 0.497451 | ko00760 |
| 18 | Riboflavin metabolism | 2 (0.87%) | 14 (0.23%) | 0.09949 | 0.497451 | ko00740 |
| 19 | Vitamin B6 metabolism | 2 (0.87%) | 15 (0.25%) | 0.111982 | 0.530442 | ko00750 |
| 20 | Glucosinolate biosynthesis | 1 (0.43%) | 4 (0.07%) | 0.145664 | 0.595498 | ko00966 |
| 21 | Thiamine metabolism | 2 (0.87%) | 19 (0.32%) | 0.165281 | 0.595498 | ko00730 |
| 22 | Folate biosynthesis | 2 (0.87%) | 19 (0.32%) | 0.165281 | 0.595498 | ko00790 |
| 23 | alpha-Linolenic acid metabolism | 4 (1.73%) | 56 (0.94%) | 0.168906 | 0.595498 | ko00592 |
| 24 | Porphyrin and chlorophyll metabolism | 4 (1.73%) | 57 (0.95%) | 0.176548 | 0.595498 | ko00860 |
| 25 | Pentose phosphate pathway | 4 (1.73%) | 57 (0.95%) | 0.176548 | 0.595498 | ko00030 |
| 26 | Alanine, aspartate and glutamate metabolism | 4 (1.73%) | 57 (0.95%) | 0.176548 | 0.595498 | ko00250 |
| 27 | Flavone and flavonol biosynthesis | 1 (0.43%) | 5 (0.08%) | 0.178649 | 0.595498 | ko00944 |
| 28 | Homologous recombination | 5 (2.16%) | 79 (1.32%) | 0.188496 | 0.605881 | ko03440 |
| 29 | Nitrogen metabolism | 3 (1.3%) | 41 (0.68%) | 0.209393 | 0.637452 | ko00910 |
| 30 | Circadian rhythm - plant | 4 (1.73%) | 62 (1.04%) | 0.21638 | 0.637452 | ko04712 |
| 31 | ABC transporters | 3 (1.3%) | 42 (0.7%) | 0.219567 | 0.637452 | ko02010 |
| 32 | Plant hormone signal transduction | 15 (6.49%) | 318 (5.31%) | 0.244965 | 0.688964 | ko04075 |
| 33 | SNARE interactions in vesicular transport | 3 (1.3%) | 49 (0.82%) | 0.292949 | 0.798952 | ko04130 |
| 34 | One carbon pool by folate | 2 (0.87%) | 29 (0.48%) | 0.308787 | 0.817378 | ko00670 |
| 35 | Terpenoid backbone biosynthesis | 4 (1.73%) | 75 (1.25%) | 0.32827 | 0.844122 | ko00900 |
| 36 | Glycine, serine and threonine metabolism | 5 (2.16%) | 100 (1.67%) | 0.342633 | 0.855158 | ko00260 |
| 37 | Limonene and pinene degradation | 1 (0.43%) | 11 (0.18%) | 0.351565 | 0.855158 | ko00903 |
| 38 | Diterpenoid biosynthesis | 3 (1.3%) | 60 (1%) | 0.40994 | 0.970094 | ko00904 |
| 39 | Carotenoid biosynthesis | 3 (1.3%) | 61 (1.02%) | 0.420374 | 0.970094 | ko00906 |
| 40 | Sulfur metabolism | 2 (0.87%) | 40 (0.67%) | 0.460668 | 0.999769 | ko00920 |
| 41 | Stilbenoid, diarylheptanoid and gingerol biosynthesis | 2 (0.87%) | 42 (0.7%) | 0.486253 | 0.999769 | ko00945 |
| 42 | Ether lipid metabolism | 2 (0.87%) | 42 (0.7%) | 0.486253 | 0.999769 | ko00565 |
| 43 | Photosynthesis | 3 (1.3%) | 68 (1.14%) | 0.491414 | 0.999769 | ko00195 |
| 44 | Glycerophospholipid metabolism | 5 (2.16%) | 128 (2.14%) | 0.553735 | 0.999769 | ko00564 |
| 45 | Inositol phosphate metabolism | 3 (1.3%) | 77 (1.29%) | 0.575986 | 0.999769 | ko00562 |
| 46 | Fructose and mannose metabolism | 3 (1.3%) | 79 (1.32%) | 0.593558 | 0.999769 | ko00051 |
| 47 | Starch and sucrose metabolism | 10 (4.33%) | 283 (4.73%) | 0.659939 | 0.999769 | ko00500 |
| 48 | Ubiquinone and other terpenoid-quinone biosynthesis | 2 (0.87%) | 59 (0.99%) | 0.670991 | 0.999769 | ko00130 |
| 49 | Phenylalanine, tyrosine and tryptophan biosynthesis | 2 (0.87%) | 59 (0.99%) | 0.670991 | 0.999769 | ko00400 |
| 50 | Glycolysis / Gluconeogenesis | 6 (2.6%) | 175 (2.92%) | 0.674774 | 0.999769 | ko00010 |
| 51 | 2-Oxocarboxylic acid metabolism | 2 (0.87%) | 60 (1%) | 0.679993 | 0.999769 | ko01210 |
| 52 | Glycerolipid metabolism | 3 (1.3%) | 91 (1.52%) | 0.68878 | 0.999769 | ko00561 |
| 53 | Fatty acid biosynthesis | 2 (0.87%) | 63 (1.05%) | 0.7058 | 0.999769 | ko00061 |
| 54 | Glyoxylate and dicarboxylate metabolism | 3 (1.3%) | 94 (1.57%) | 0.709803 | 0.999769 | ko00630 |
| 55 | Peroxisome | 3 (1.3%) | 99 (1.65%) | 0.742401 | 0.999769 | ko04146 |
| 56 | Arginine biosynthesis | 1 (0.43%) | 36 (0.6%) | 0.758469 | 0.999769 | ko00220 |
| 57 | Fatty acid metabolism | 3 (1.3%) | 104 (1.74%) | 0.772035 | 0.999769 | ko01212 |
| 58 | Phenylalanine metabolism | 2 (0.87%) | 74 (1.24%) | 0.785986 | 0.999769 | ko00360 |
| 59 | Steroid biosynthesis | 1 (0.43%) | 40 (0.67%) | 0.793852 | 0.999769 | ko00100 |
| 60 | Proteasome | 2 (0.87%) | 76 (1.27%) | 0.79832 | 0.999769 | ko03050 |
| 61 | Protein export | 2 (0.87%) | 76 (1.27%) | 0.79832 | 0.999769 | ko03060 |
| 62 | Tryptophan metabolism | 1 (0.43%) | 41 (0.68%) | 0.801859 | 0.999769 | ko00380 |
| 63 | Pentose and glucuronate interconversions | 4 (1.73%) | 142 (2.37%) | 0.804815 | 0.999769 | ko00040 |
| 64 | Biosynthesis of unsaturated fatty acids | 1 (0.43%) | 43 (0.72%) | 0.816957 | 0.999769 | ko01040 |
| 65 | Sphingolipid metabolism | 1 (0.43%) | 43 (0.72%) | 0.816957 | 0.999769 | ko00600 |
| 66 | Zeatin biosynthesis | 1 (0.43%) | 44 (0.73%) | 0.82407 | 0.999769 | ko00908 |
| 67 | Phosphatidylinositol signaling system | 2 (0.87%) | 85 (1.42%) | 0.846365 | 0.999769 | ko04070 |
| 68 | Cysteine and methionine metabolism | 3 (1.3%) | 122 (2.04%) | 0.856441 | 0.999769 | ko00270 |
| 69 | Fatty acid degradation | 1 (0.43%) | 53 (0.89%) | 0.876895 | 0.999769 | ko00071 |
| 70 | Nucleotide excision repair | 2 (0.87%) | 95 (1.59%) | 0.887409 | 0.999769 | ko03420 |
| 71 | RNA degradation | 3 (1.3%) | 132 (2.2%) | 0.890436 | 0.999769 | ko03018 |
| 72 | Carbon metabolism | 9 (3.9%) | 338 (5.65%) | 0.91288 | 0.999769 | ko01200 |
| 73 | Ascorbate and aldarate metabolism | 1 (0.43%) | 62 (1.04%) | 0.913906 | 0.999769 | ko00053 |
| 74 | Spliceosome | 6 (2.6%) | 244 (4.08%) | 0.916019 | 0.999769 | ko03040 |
| 75 | Fatty acid elongation | 1 (0.43%) | 64 (1.07%) | 0.920489 | 0.999769 | ko00062 |
| 76 | Mismatch repair | 1 (0.43%) | 65 (1.09%) | 0.92359 | 0.999769 | ko03430 |
| 77 | Arginine and proline metabolism | 1 (0.43%) | 66 (1.1%) | 0.926571 | 0.999769 | ko00330 |
| 78 | Ubiquitin mediated proteolysis | 3 (1.3%) | 151 (2.52%) | 0.935862 | 0.999769 | ko04120 |
| 79 | DNA replication | 1 (0.43%) | 77 (1.29%) | 0.95262 | 0.999769 | ko03030 |
| 80 | Aminoacyl-tRNA biosynthesis | 1 (0.43%) | 79 (1.32%) | 0.956252 | 0.999769 | ko00970 |
| 81 | Biosynthesis of amino acids | 6 (2.6%) | 274 (4.58%) | 0.958172 | 0.999769 | ko01230 |
| 82 | Ribosome biogenesis in eukaryotes | 2 (0.87%) | 128 (2.14%) | 0.961564 | 0.999769 | ko03008 |
| 83 | Galactose metabolism | 1 (0.43%) | 83 (1.39%) | 0.962704 | 0.999769 | ko00052 |
| 84 | Pyrimidine metabolism | 2 (0.87%) | 133 (2.22%) | 0.967513 | 0.999769 | ko00240 |
| 85 | Pyruvate metabolism | 2 (0.87%) | 135 (2.25%) | 0.969636 | 0.999769 | ko00620 |
| 86 | Purine metabolism | 3 (1.3%) | 187 (3.12%) | 0.978195 | 0.999769 | ko00230 |
| 87 | Endocytosis | 3 (1.3%) | 202 (3.37%) | 0.98637 | 0.999769 | ko04144 |
| 88 | Phagosome | 1 (0.43%) | 112 (1.87%) | 0.988311 | 0.999769 | ko04145 |
| 89 | Ribosome | 8 (3.46%) | 420 (7.02%) | 0.993607 | 0.999769 | ko03010 |
| 90 | RNA transport | 1 (0.43%) | 209 (3.49%) | 0.999769 | 0.999769 | ko03013 |
